# The effect of different surface treatment on shear bond strength of soft and hard liners to CAD-CAM and conventional denture base resins: *in vitro* comparative study

**DOI:** 10.3389/fdmed.2026.1736153

**Published:** 2026-02-16

**Authors:** Zainab Albazroun, Fatimah A. Aldobais, Sarah Aldehaileb, Atheer Alabdullatif, Safiyah Almahdi, Aminah M. Alsayoud, Faisal D. Al-Qarni, Ahmed Alshareef, Sultan Akhtar, Mohammed M. Gad

**Affiliations:** 1 College of Dentistry, Imam Abdulrahman Bin Faisal University, Dammam, Saudi Arabia; 2 Department of Substitutive Dental Sciences, College of Dentistry, Imam Abdulrahman Bin Faisal University, Dammam, Saudi Arabia; 3 Department of Biophysics, Institute for Research and Medical Consultations (IRMC), Imam Abdulrahman Bin Faisal University, Dammam, Saudi Arabia

**Keywords:** CAD-CAM, denture, denture liner, shear bond strength, surface treatment

## Abstract

**Purpose:**

To evaluate the impact of different surface treatments on shear bond strength (SBS) of hard and soft denture liners bonded to CAD-CAM and conventional denture base resins.

**Methods:**

A total of 300 acrylic denture base specimens were fabricated with dimensions of 10 × 10 × 3 mm, with five different denture base materials: two milled (AvaDent and IvoCad), two printed (NexDent and FormLabs), and one heat-processed acrylic resin. The specimens underwent 5,000 thermal cycles both before and after the reline procedures, and each specimen was treated using one of three surface treatment methods: sandblasting, bur roughening, or no treatment (control). The reline procedure was performed with either a soft liner or a hard liner (*n* = 10). Shear bond strength was tested using a universal testing machine with a crosshead speed of 1 mm/min until failure. Data were analyzed by two-way ANOVA (*α* = 0.05).

**Results:**

Sandblasting significantly increases the SBS of hard liners to AvaDent, IvoCad, NextDent, and conventional resins compared to both the control and bur roughening groups (*p* ≤ 0.0001). For FormLabs, sandblasting significantly improved SBS compared to the control group only (*p* ≤ 0.01). In the case of soft liners, bur roughening significantly enhanced SBS for AvaDent, IvoCad, and conventional resins, while no significant improvement was observed for NextDent and FormLabs.

**Conclusion:**

Sandblasting is recommended to enhance the shear bond strength of hard liners across various denture base resins, whereas bur roughening is more effective when using soft liners.

## Introduction

One of the primary goals in fabricating complete dentures is to achieve the optimal fit of the denture base to the residual ridges. When a denture is worn for a long period of time, gaps may be formed between the denture fitting surface and the mucosal membrane (1). A reline procedure is required when gradual changes of oral tissues occur, for the purpose of improving denture adaptation and stability (2). Resilient lining materials have been relied on to create a cushion between the denture base and the supporting tissues, distributing the pressures delivered to soft tissues during function and facilitating more harmonious stress distribution at the tissue/lining interface (3, 4).

The two forms of resilient denture lining materials are silicone elastomers and plasticized acrylic resins. Both types are available in either auto-polymerizing or heat-curing forms, with varieties in the percentage of plasticizers (5). Acrylic resin-based resilient denture liners frequently have plasticizers that may leach out of the material, causing hardening of the liner over time. The polymer silicone-based resilient lining material is an elastomer that does not require an external plasticizer, making it more stable over time (6).

Shear bond strength is the ability of a material or component to withstand failure when subjected to shear force (7). Shear force, applied at the denture/reline interface, is considered more effective than tensile load, as it better simulates the multidirectional stresses occurring during mastication (8). It has also been widely used in previous research to evaluate the bond strength between denture base and reline materials (9–11). Various approaches have been investigated to enhance shear bond strength, such as surface treatment (10, 12, 13). Despite these efforts, researchers reported a weak bond at the resin/reline interface characterized by adhesive failure (10).

According to a recent review, the bonding strength of milled CAD-CAM denture base resins and relining materials is comparable to that of conventional denture base resins, irrespective of the denture lining material's consistency. Conversely, the bond strength of the milled denture base was stronger than that of the 3D-printed CAD-CAM resin (14). Li et al. (15) discovered that the type of denture base material and the type of soft relining material utilized have an impact on the tensile bond strength of soft liners to PMMA, milled, and 3D-printed resins. Alfaraj et al. (16) stated that, when compared to denture base resins, heat-polymerized and auto-polymerized relining produced the strongest bond, whilst 3D-printed resins reinforced with auto-polymerized hard relining produced the weakest bond. Surface treatment was recommended to overcome the weak bone strength (14, 17, 18).

Before performing a reline on dentures, most manufacturers recommend that the denture fitting surface be adjusted (6). Various surface treatments, including bur roughening, airborne particle abrasion, monomer application, and airborne particle abrasion combined with bonding agents, were used to enhance the bond strength (8, 19). Sandblasting is a technique that uses alumina particles of a variety of sizes to roughen surfaces (8). Surface changes created by the alumina abrasive particles increase surface bonding area, resulting in an enhanced bond strength (20). A 3D-printed denture base resin treated with 50 μm airborne particle abrasion exhibits better bond strength (17). Similarly, surface modifications using bur grinding aim to increase surface area and improve mechanical retention (6). Depending on the surface treatment method, the shear bond strength of the 3D-printed denture base and the chairside relining material varied noticeably (21). Mechanical surface treatments and universal adhesive applications are more effective in maintaining adhesion across all production techniques (17). The specific type of relining material, surface treatment method, and denture base materials all determine bonding strength and should be considered when selecting relining materials that will cooperate harmoniously with denture base types (9, 18). Limited papers exist that compare hard and soft reline materials with different surface treatments to conventional, CAD-CAM milled, and 3D-printed denture bases.

A few studies investigated the bond strength of hard and soft reline materials with CAD/CAM milled and 3D printed denture base resin. Additionally, there are lack of studies investigated the effect of bur roughening surface treatment in comparison with air-abrasive treatment method. Therefore, the objective of this study is to investigate how mechanical roughening techniques, specifically bur roughening and sandblasting, affect the shear bond strength of hard and soft reline materials when applied to various denture base resins.

## Materials and methods

### Specimens preparation

To calculate the sample size, an online power analysis calculator was used. The mean and standard deviation values were obtained from a previously published study (8). The statistical power was set at 80%, and the level of significance was set at 0.05. Based on these parameters, the calculated sample size indicated the need for ten specimens per group, resulting in a total of 300 specimens were used to test the SBS. In this study, five distinct denture base materials were evaluated: two CAD/CAM milled resins, AvaDent and IvoCad; two 3D-printed resins, FormLabs and NextDent; and one heat-polymerized resin: Major Base 20 was used in this study with one hard denture liner (Ufi Gel Hard C) and one soft liner (KEYSTONE Versacryl Reline Kit—Self Cure Soft Reline) (Table 1).

**Table 1:** Summary of materials used in present study and fabrication methods.

Materials (Brand name)	Composition	Specimens fabrication method
Heat-polymerized acrylic resin (HP) (Major Base.20, Major Prodotti Dentari Spa, Momcalieri, Italy)	Powder: Polymer (PMMA) þ initiator [benzoyl peroxide (BPO)] (0.5%) þ pigments (salts of cadmium or iron or organic dyes) Liquid: Monomer (MMA) þ cross-linking agent [Ethylene glycol dimethacrylate (EGDMA) 10%] þ inhibitor (hydroquinone	Conventional heat polymerization method Polymerization cycle: 90 min in a water bath by heating to 74 °C, then 100 °C for 30 min
IvoCad (IvoCad (Ivoclar Vivadent, Schaan, Liechtenstein)	Prepolymerized PMMA discs 50%–100% methyl methacrylate 2.5%–10% 1,4-butanediol dimethacrylate	Cut from pre-polymerized acrylic disc using diamond saw (Isomet 5000 Linear Precision Saw, Buehler Ltd, Bluff, IL)
AvaDent (AvaDent Digital Dental Solutions, Scottsdale, AZ, USA)	Prepolymerized PMMA (PMMA 99.5%, pigments <1.0%)	
NextDent Denture 3D+ NextDent B.V., Soesterberg, The Netherlands	Ester-based monomer; Bisacylphosphine oxide (BAPO) phenylbis (2, 4, 6-trimethylbenzoyl)-phosphine oxide (Omnirad 819)	3D printed specimens Technology: Printer: NextDent 5100 Printing layer thickness: 50µm Printing orientation: 0-degree Post-curing machine: LC-D Print Box Post-curing time/temp.: 30 min/60 °C
Formlabs Denture Base Resin LP Formlabs Inc., Somerville, MA, USA	55%–75% w/w urethane dimethacrylate, 15%–25% w/w methacrylate monomers, and < 0.9% w/w phenyl bis(2,4,6-trimethylbenzoyl)-phosphine oxide	3D printed specimens Technology: Printer: Form 2 Printing layer thickness: 50 µm Printing orientation: 0-degree Post-curing machine: FormCure Post-curing time/temp.: 30 min/ 60 °C
Ufi Gel Hard C	Hydroxyethyl methacrylate Initiators (benzoyl peroxide) Acetone Pigments	1:1 cartridge
KEYSTONE Versacryl Reline Kit—Self Cure Soft Reline	Bis(2-ethylhexyl) phthalate N.Ndimethyl-p-toluidine di-n-butyl phthalate	Liquid ratio: 1.5-parts powder to 1-part liquid by weight

Acrylic denture bases were prepared in dimensions of 10 × 10 × 3 mm according to the manufacturer's recommendations. All specimens were standardized in terms of the polishing method and were examined by a single investigator. The prepared specimens were polished using silicon carbide abrasive papers available in various grits (500, 800, 1,200) that were mounted on an automated polishing machine (Metaserve 250 grinder-polisher; Buehler, Lake Bluff, IL, USA).

Each specimen was embedded in self-polymerizing acrylic resin with one surface completely exposed using a custom-made, cylindrical-shaped silicon mold measuring 19 × 10 mm. Following polymerization, the exposed surfaces of the specimens were carefully examined, and surface irregularities were eliminated.

Then, the specimens were subjected to 5,000 thermal cycles before the relining procedure to simulate aged denture bases, and after the relining procedure to evaluate the shear bond strength between the reline material and the denture base, 5 °C–55 °C with a 30 s dwell time within a thermocycling machine (Thermocycler, THE-1100/THE-1200, SD Mechatronik GMBH Miesbacher, Westerham Germany).

### Surface treatment

The specimens were divided into two major groups based on the type of reline materials (*n* = 150). Each acrylic resin base was further divided into three groups according to the surface treatment: the first group was without surface treatment (control); the second group was treated with sandblasting with 250 μm aluminum oxide particles (SB) using a sandblasting machine (Wassermann Dental Machine, GmbH, Hamburg, Germany) from 1 cm distance for 10 s with 3.5 bar pressure. A customized metal jig with an inner diameter of 6 mm was used to determine the area of sandblasting application (8). The third group underwent surface roughening using a blue-coded tungsten carbide bur. The bur was applied in one direction under light manual pressure, with approximately 12 unidirectional strokes to standardize the surface treatment. In the case of soft liners, the bonding agent was applied in accordance with the manufacturer's recommendations. Two layers of bonding agent were applied to the denture base, with a drying time of 5 min between each layer.

### Relining procedure

**Figure 1:**
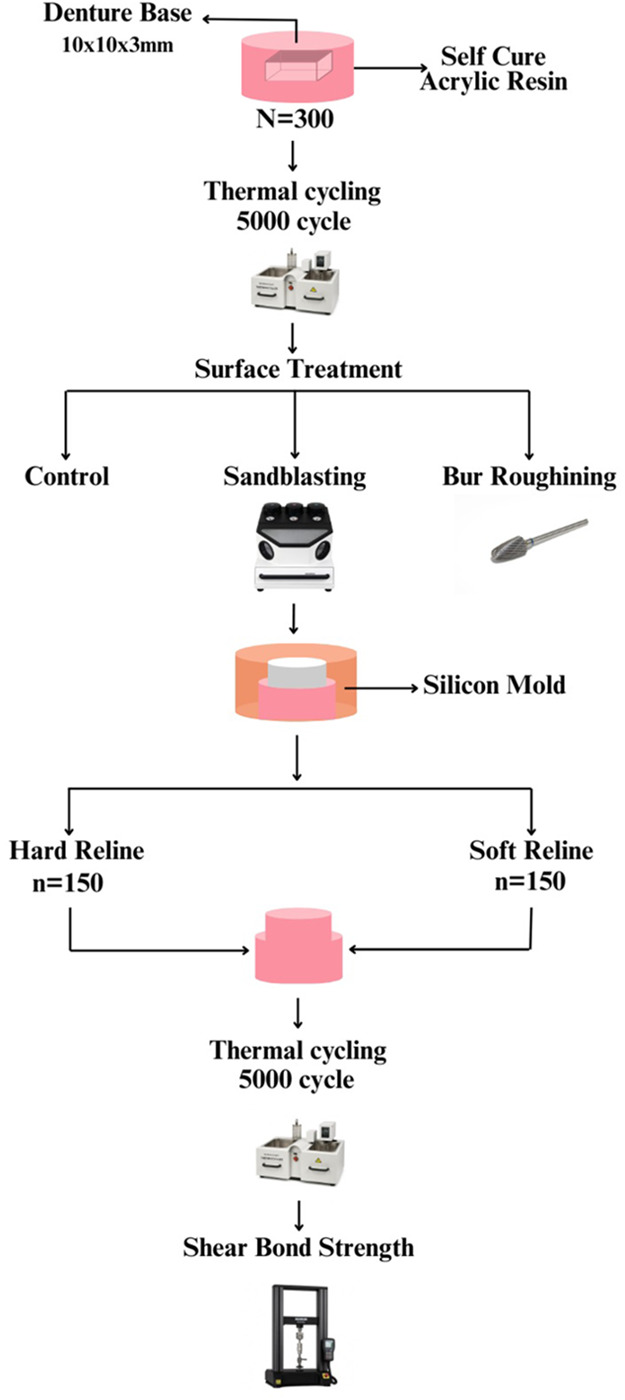
Illustration of study methodology.

As described in the previous study (10), At the centre of the prepared denture base specimen, a metal cylinder (4 mm diameter × 6 mm length) was fixed within the holder. A customized mold was prepared, into which each specimen was fixed. The reline materials was then mixed and packed in the space created by the cylinder and covered with a glass slap under pressure (1 kg) for 15 min. After polymerization, the specimens were stored in distilled water (37 °C) for two days. Subsequently, the specimens were subjected to 5,000 thermal cycles prior to shear bond strength testing (Figure 1).

### Shear bond strength

**Figure 2:**
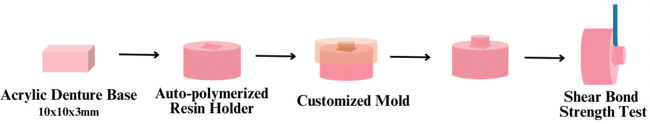
Illustration of specimen preparation and shear bond strength test.

The shear bond strength values of the reline specimens were evaluated using a universal testing machine (Instron, Instron Corp., Norwood, MA, USA). Each specimen was mounted onto a specially designed jig, and a chisel (knife-edge shear type) was used to apply a shear load parallel to the bonded interface at a crosshead speed of 1 mm/min until failure occurred (Figure 2). SBS was calculated using the (MPa) = F/A, where *F* is the force at failure in newtons (N), and *A* is the bonding area in square millimetres (mm^2^).

### Statistical analysis

Normality of the data was checked using the Shapiro–Wilk test and was confirmed to be normally distributed. The statistical analysis was processed using one-way ANOVA and an unpaired two-way ANOVA (GraphPad Prism software). Tukey's *post hoc* test was used for pairwise comparisons. *P*-value is set at <0.05 (* if *P* ≤ 0.05, ** if *P* ≤ 0.01, *** if *P* ≤ 0.001, **** if *P* ≤ 0.0001) is considered statistically significant.

## Results

### SEM effect of surface treatment

**Figure 3:**
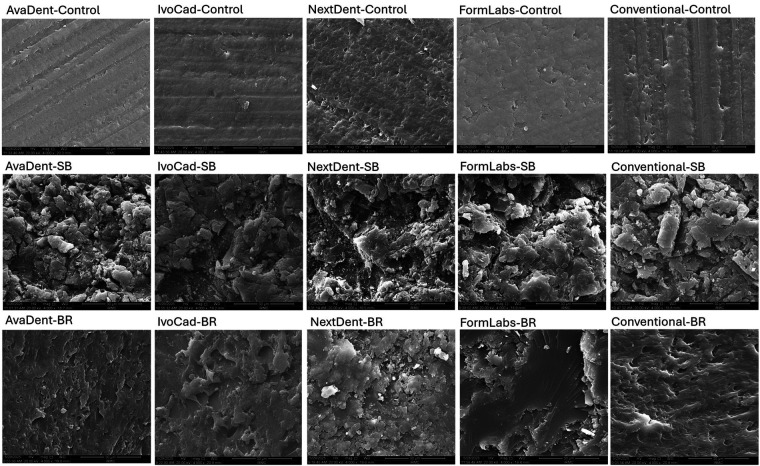
Different surface treatment under SEM under ×30.

Figure 3 displays representative SEM images illustrating the effect of surface treatments on various denture base resins. The specimens without surface treatment (1st raw) showed a smooth surface with minor irregularities and some striations representing the printing layer and/or polishing grits The specimens’ surfaces underwent a dramatic change in surface topography following SB treatment (2nd row). SB resulted in more irregularities in all treated specimens. While bur roughening (3rd raw) resulted in some roughening, it was localized, uneven, and shallower than the SB effect.

### Hard liner

**Figure 4:**
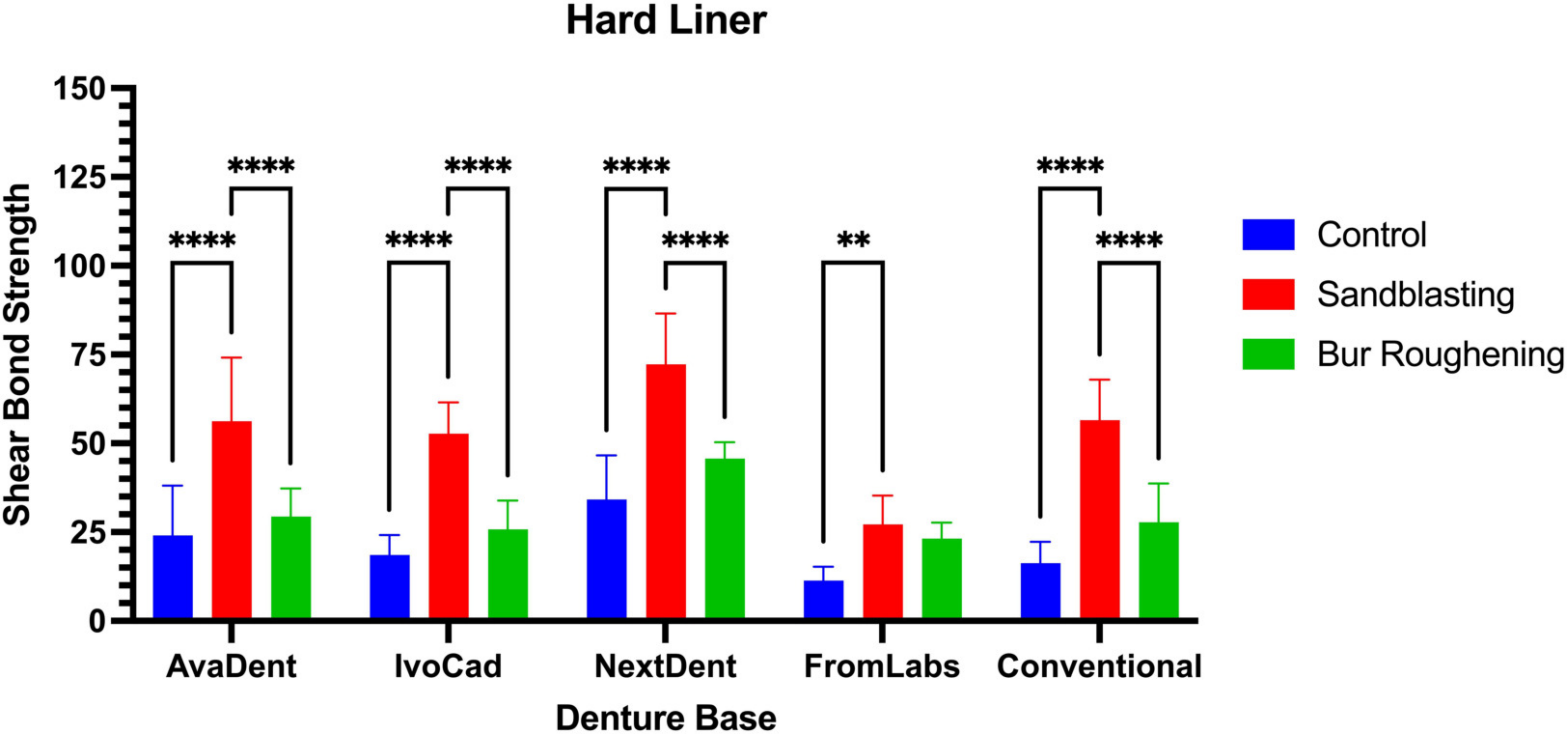
SBS of hard liner with different denture base resin and surface treatment.

Using sandblasting as a surface treatment with milled denture base resins (AvaDent and IvoCad) resulted in a statistically significant increase in SBS compared to both the control group and bur roughening (*p* ≤ 0.0001). Additionally, sandblasting applied to the 3D-printed NextDent resin exhibits the highest SBS among all groups in hard liner 72.25 MPa (Table 2; Figure 4) with statistically significant differences compared to both the control and bur roughening groups (*p* ≤ 0.0001). For FormLabs, sandblasting significantly increased SBS compared to the control group (*p* ≤ 0.01), but no significant difference was observed when compared to bur roughening; the lowest SBS was recorded in the control group of FormLabs at 11.4 MPa. Similarly, the conventional denture base resin treated with sandblasting demonstrated a significant increase in SBS compared to both the control and bur roughening groups (*p* ≤ 0.0001).

**Table 2:** Mean values, SD, and significance in SBS of hard liner .

Denture base resin	Code	Surface treatment			*P*
		Control	Sandblasting	Bur Roughening	
Milled	AvaDent	24.04 (12.4)^A^	56.22 (15.8)^A,B,a,b^	29.4 (6.95)^B,a^	0.0001****
	IvoCad	18.6 (4.9)^A,a^	52.78 (7.7)^A,B,c,d^	25.8 (7.11)^B,b^	0.0001****
3D printed	NextDent	34.24 (10.88)^A,a,b,c^	72.25 (12.5)^A,B,a,c,e,f^	45.66 (4.14)^B,a,b,c,d^	0.0001****
	FormLabs	11.4 (3.45)^A,b^	27.17 (7.1)^A,b,d,e,g^	23.18 (3.96)^c^	0.0063**
Conventional		16.27 (5.3)^A,c^	56.54 (10)^A,B,f,g^	27.79 (9.6)^B,d^	0.0001****
P		0.0003***	0.0001****	0.0001****	

Statistically significant at **if *P* ≤ 0.01, ***if *P* ≤ 0.001, ****if *P* ≤ 0.0001.

Same small alphabets showed the significant difference in each column.

Same capital alphabets in each row showed significant difference.

The combined effect of denture base material and surface treatment on shear bond strength was analyzed using two-way ANOVA. Analysis revealed a significant interaction between the two factors (*p* = 0.0080) (Table 3).

**Table 3:** Two-way ANOVA results of SBS of hard liner .

Source	Type III sum of squares	Df	Mean square	F—Test	*P*
Material	11,182	4	2,796	27.57	*P* < 0.0001****
Surface treatment	21,738	2	10,869	107.2	*P* < 0.0,001****
Material * Surface treatment	2252	8	281.6	2.777	*P* = 0.0080***

Statistically significant at ***if *P* ≤ 0.001, ****if *P* ≤ 0.0001.

### Nature of failure

**Figure 5:**
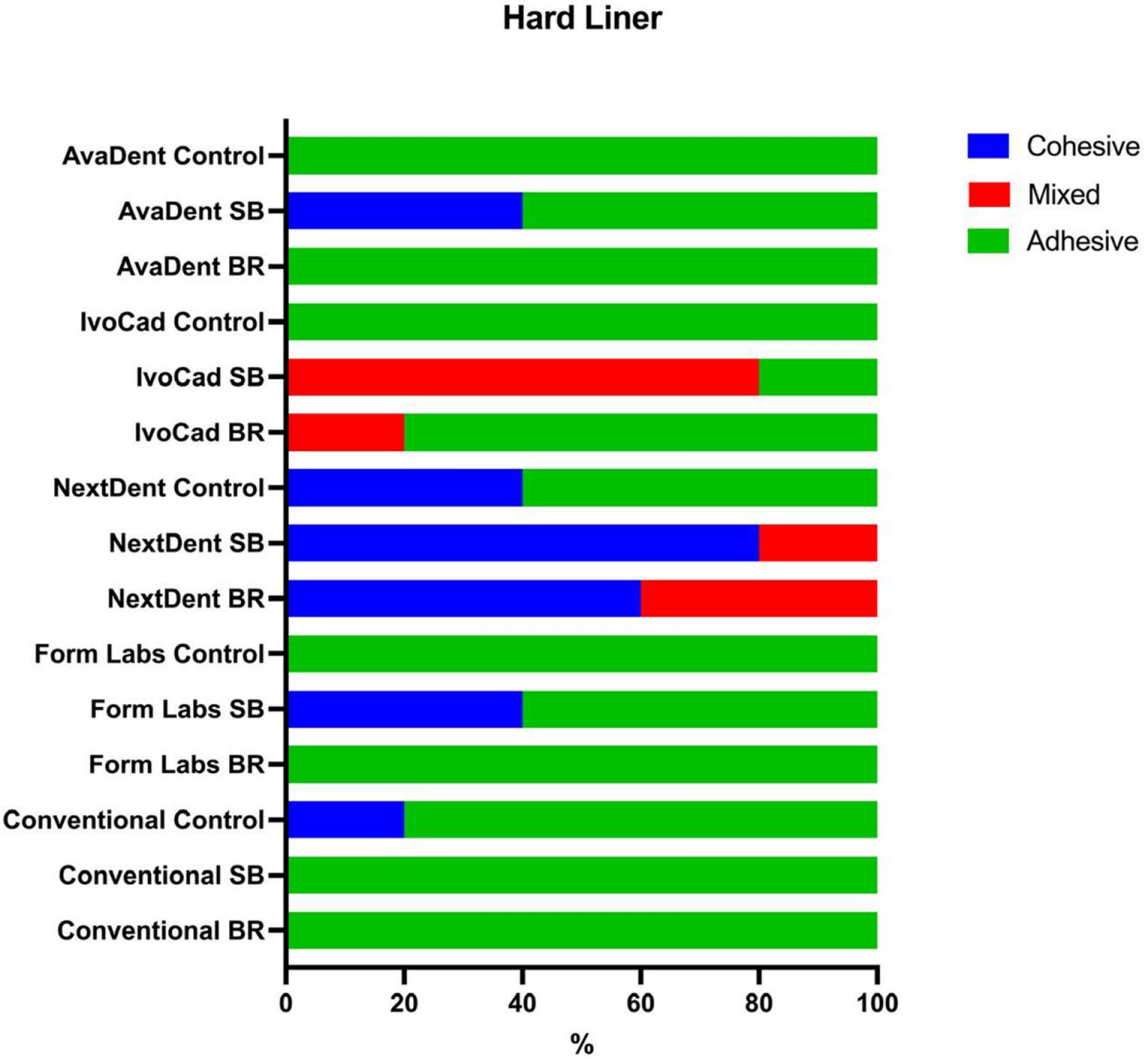
Show nature of failure in percentages for hard liner.

**Figure 6:**
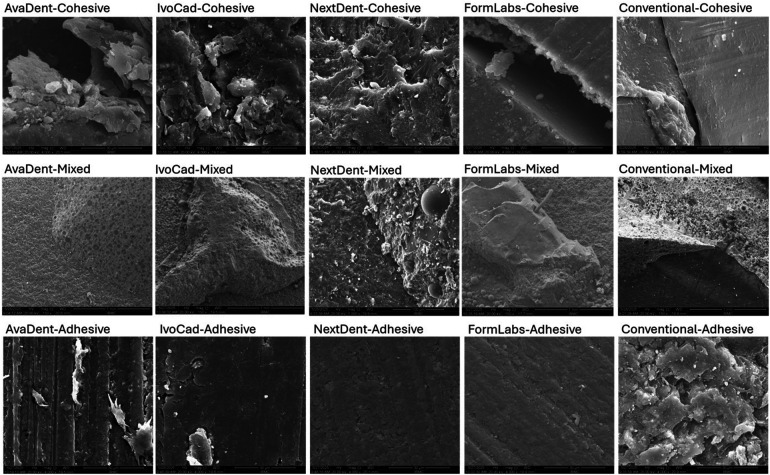
Representatives SEM images show the nature of failure with SEM under ×1–×30.

In this study, the nature of the fracture was analyzed in terms of adhesive, cohesive, or mixed, as described in previous studies. Regarding the nature of failure, adhesive failure was the dominant type in the case of the control group, which accounted for 100% of failures with Avadent, IvoCad, and FormLabs. The other groups subjected to testing demonstrated cohesive and mixed failure modes subsequent to surface treatment, notably with the SB treatment, which exhibited the highest percentage of the previously mentioned types (Figures 5, 6).

### Soft liner

**Figure 7:**
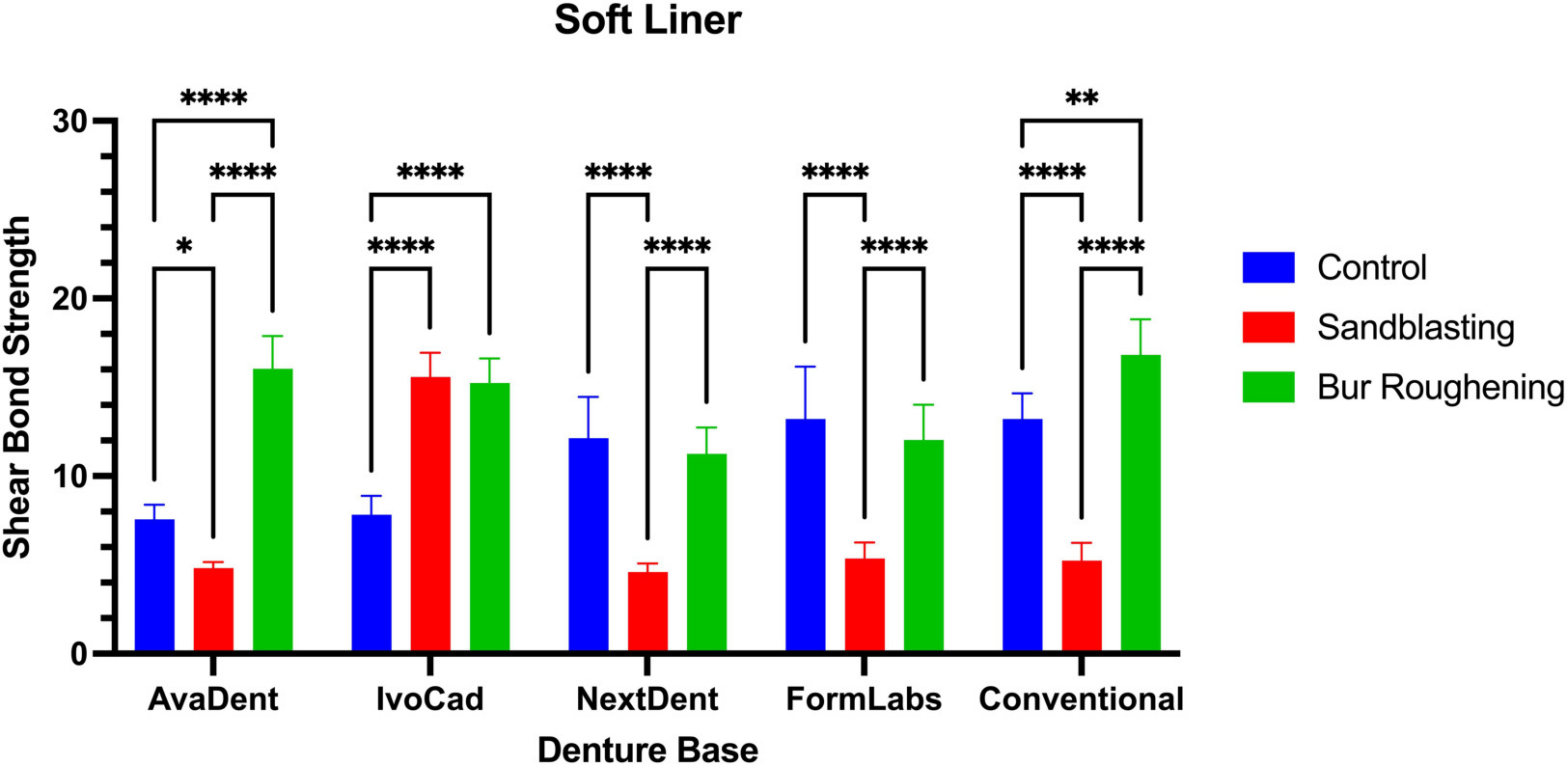
Show the SBS of soft liner with different surface treatment and denture base resins.

The mean and standard deviation (SD) of the shear bond strength for different denture base resins relined with a soft liner are presented in (Figure 7; Table 4). Bur roughening significantly increased the SBS for AvaDent and IvoCad resins compared to the control group (*P* < 0.0001). However, no significant difference in SBS was found between the bur-roughened and control groups for NextDent (ND) and FormLabs (FL). Conventional denture base resins treated with bur roughening showed significantly higher SBS than the control group (*P* ≤ 0.01), with the highest SBS value recorded across all soft liner groups (16.82 MPa).

**Table 4:** Mean values, SD, and significance in SBS of soft liner.

Denture base resin	Code	Surface treatment			*P*-value
		Control	Sandblasting	Bur roughening	
Milled	AvaDent	7.57 (0.73)^A,B,a,b,c^	4.82 (0.29)^A,C,a^	16.03 (1.66)^B,C,a,b^	0.0001****
	IvoCad	7.82 (0.96)^A,B,d,e,f^	15.57 (1.22)^A,a,b,c,d^	15.25 (1.21)^B,c,d^	0.0001****
3D printed	NextDent	12.14 (2.07)^A,a,d^	4.60 (0.42)^A,B,b^	11.25 (1.33)^B,a,c,e^	0.0001****
	FormLabs	13.21 (2.64)^A,b,e^	5.35 (0.80)^A,B,c^	12.03 (1.78)^B,b,d,f^	0.0001****
Conventional	13.2 (1.28)^A,B,c,f^	5.25 (0.87)^A,C,d^	16.82 (1.79)^B,C,e,f^	0.0001****	
*P*-value	0.0001****	0.0001****	0.0001****		

Statistically significant at ****if *P* ≤ 0.0001.

Same small alphabets showed the significant difference in each column.

Same capital alphabets in each row showed significant difference.

In contrast, sandblasting significantly reduced the SBS of AvaDent, NextDent, FormLabs, and conventional resins when compared to both the control and bur-roughened groups. However, sandblasting resulted in a significant increase in SBS for IvoCad (*P* < 0.0001).

The combined effect of denture base material and surface treatment on shear bond strength bonded to soft liner was analyzed using two-way ANOVA. The analysis showed a statistically significant interaction between the material and surface treatment (*P* < 0.0001), as shown in (Table 5).

**Table 5:** Two-way ANOVA results for soft liner.

Source	Type III sum of squares	df	Mean Square	F—Test	*P*
Material	142.4	4	35.60	14.23	*P* < 0.0001****
Surface treatment	640.5	2	320.2	128.0	*P* < 0.0001****
Material * Surface treatment	593.0	8	74.13	29.63	*P* < 0.0001****

Statistically significant at ****if *P* ≤ 0.0001.

### Nature of failure

**Figure 8:**
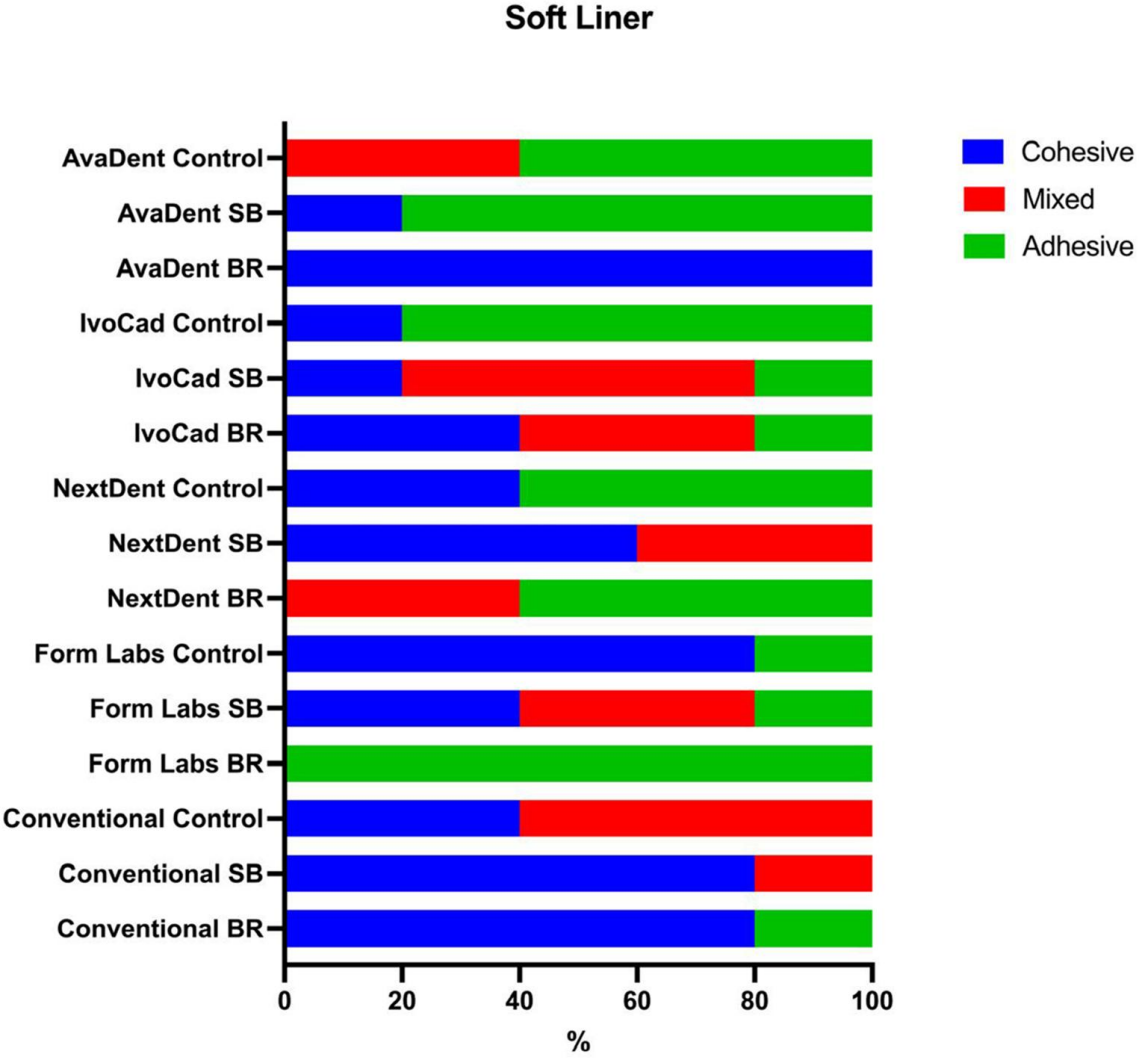
Show nature of failure in percentages for soft liner.

Cohesive failure was the predominant failure mode observed with the soft liner after bur roughening, accounting for 100% of failures in the AvaDent group and 80% in the conventional denture base group. Additionally, when sandblasting was used, cohesive and mixed failures were frequently observed in both the IvoCad and conventional groups (Figures 6, 8).

## Discussion

The primary objective of this study was to evaluate the shear bond strength of CAD-CAM milled, 3D-printed, and conventional denture base resins following relining with either soft or hard liners with various surface treatments. The SBS was significantly affected by surface treatments; hence, the null hypothesis was rejected.

Various mechanical methods, such as airborne-particle abrasion and bur roughening, have been used in studies to enhance the bonding of base materials (17). Mechanical surface treatment by sandblasting with alumina oxide particles often results in greater bond strength and surface roughness when compared to untreated and chemical surface treatments (8, 22). The microroughness improves the mechanical interlocking of materials and increases the bonding area of the surface, which strengthens the shear bonds that link the two materials (22).

Thermocycling is a laboratory method in which materials are exposed to alternating hot and cold water baths to mimic the thermal conditions of the oral cavity (23). In the oral cavity, both the denture base and the reline material experience a wide range of temperature changes (23). The alternating exposure to heat and cold leads to repeated expansion and contraction of both the tooth and denture base material, which can place stress on the bond interface and weaken the bond strength. Additionally, these thermal variations may significantly affect the mechanical properties of the materials over time (24, 25). Heat stress may increase the distance between polymer chains, leading to an increase in water sorption (26). Water sorption is a primary factor contributing to reduced bond strength, as it causes resin swelling and disrupts the bonding interface (27). This effect becomes more pronounced at elevated temperatures, where water absorption increases (28). The absorbed water acts as a plasticizer, penetrating the interface between the denture base and the reline material, ultimately compromising the bond (27, 29). Hence, in this study, all materials were subjected to thermocycling (5,000 cycles of 5 °C–55 °C) to simulate six months of denture use.

### Hard reline

In the present study, the milled denture base resins AvaDent and IvoCad demonstrated comparable responses to surface treatment when bonded with a hard reline material. Both materials exhibited a increase in SBS following sandblasting due to increasing the surface area, whereas bur roughening did not result in a significant difference compared to the control group. Similarly, the 3D-printed resins, NextDent and FormLabs, showed a significant enhancement in SBS after sandblasting, with no notable improvement observed with bur roughening. Additionally, the conventional heat-polymerized resin also exhibited a significant increase in SBS following sandblasting treatment. These results suggest that sandblasting is an effective surface treatment method for enhancing the bond strength of various denture base resins to hard reline materials, while bur roughening does not provide a comparable benefit.

Sandblasting enhances shear bond strength by eliminating surface impurities and contaminants from the denture base (30, 31). This process enhances mechanical retention by increasing both the surface area and surface roughness, thereby facilitating improved bonding (31). The roughened surface created by sandblasting promotes better adhesive penetration and mechanical interlocking, resulting in a stronger and more durable bond between the denture base and the reline material (32, 33).

Numerous studies (34, 35) have demonstrated that airborne-particle abrasion with aluminum oxide (Al_2_O_3_) significantly enhances the bond strength of polymethyl methacrylate (PMMA) denture base resins. Klaiber et al. (34) reported that treating PMMA surfaces with 250 μm Al_2_O_3_ particles at 0.3 MPa for 30 s significantly increased the bond strength to autopolymerized resin. Erbulak et al. (36) found that, among various surface treatments, abrasion with 110 μm Al_2_O_3_ at 2 MPa for 30 s produced the strongest bond with autopolymerized acrylic resin. Similarly, Li et al. (37) demonstrated that abrasion using 125 μm Al_2_O_3_ at 2 bar resulted in the highest shear bond strength (17.31 ± 5.67 MPa), concluding that mechanical surface treatment is effective. Akın et al. (35) also confirmed that airborne-particle abrasion of PMMA surfaces with Al_2_O_3_ particles improves bonding performance.

In a comparative study, Karaokutan et al. (17) evaluated subtractive, additive, and conventional heat-polymerized resin relined with autopolymerized acrylic resin. Their findings revealed that surfaces treated with 50 μm Al_2_O_3_ particles exhibited a higher bond strength than tungsten carbide bur. Similarly, our study found that sandblasting provided higher shear bond strength than bur roughening when used with a hard liner. On the other hand, Baig et al. (38) found a statistically significant difference in the SBS of relined specimens (*P* < 0.05) when various burs were used for surface preparation. However, no significant difference was observed between the standard bur and the control group. Baig et al. concluded that the type of bur used to prepare the denture base for relining may affect the surface roughness and, consequently, the reline bond strength of urethane-based dimethacrylate denture base resin (39).

Sandblasted specimens predominantly exhibited cohesive and mixed failure modes, in contrast to the adhesive failures observed in untreated controls, indicating superior bonding performance. SEM analysis confirmed increased surface irregularities following sandblasting, which probably enhanced mechanical interlocking. As a result, sandblasting significantly improved the shear bond strength of all tested denture base resins relined with a hard liner.

Based on the findings, sandblasting is recommended as an effective surface treatment for CAD/CAM milled 3D-printed, and conventional denture base resins when used with a hard liner. In contrast, bur roughening showed no significant effect on bond strength across any of the denture base types tested in this study when used in combination with a hard liner.

### Soft reline

In the present study, the milled denture base resins AvaDent and IvoCad, as well as the conventional heat-polymerized resin, exhibited higher bond strength with the soft liner when treated with bur roughening. However, NextDent and FormLabs, both 3D-printed resins, showed no improvement with bur roughening and demonstrated a negative effect when treated with sandblasting. These findings suggest that bur roughening is an effective surface treatment for improving the bond strength of various denture base resins, conventional and milled, to soft liners, whereas sandblasting does not offer a similar advantage.

The superior shear bond strength observed with PMMA may be attributed to the chemical compatibility between acrylic-based soft liners and conventional denture base materials, largely due to the similarity in their chemical structures (39–41). In contrast, 3D-printed denture bases are composed of light-polymerizable resins that contain additional additives to facilitate photopolymerization. These materials require curing in specialized light-curing units, a process that can alter the surface energy of the printed denture base (41, 42). This explains why 3D-printed denture bases tend to exhibit lower SBS compared to PMMA.

Silicone-based denture liners demonstrate superior mechanical strength and durability compared to resin-based liners; however, they suffer from poor chemical bonding capabilities, with adhesion failures often linked to the bonding agent used (43, 44). Studies have shown that adhesive failure rates between silicone liners and PMMA denture bases can increase significantly, from 13.8% to 60%—after 30 days of water storage indicating a progressive decline in bond strength over time (45). In our study, the aging process was simulated over a six-month period of use, which correlated with a reduction in bond strength. Efforts to improve adhesion using silica air abrasion and salinization were unsuccessful, as the surface roughness generated by 30 μm particles did not permit sufficient liner penetration into the acrylic base. However, mechanical surface roughening by bur may provide better penetration, potentially explaining the relatively high mechanical strength observed despite the material's weak chemical bonding.

Minami et al. (46) reported that roughening the denture base resin surface using air-particle abrasion did not effectively enhance the failure load or improve the long-term bond durability of a soft denture liner to the denture base. Atsü et al. (47) found that sandblasting surface treatments applied to the denture base resin did not improve the tensile bond strength of a silicone-based soft liner to heat-cured acrylic resin. In a systematic review, Özdemir et al. (48) reported that although sandblasting was frequently used as a surface treatment for resilient lining materials, it was ineffective in enhancing bond strength. Due to the limited existing research on the use of bur roughening in combination with soft liners, direct comparisons have been challenging. This study contributes to addressing the existing gap in the literature by demonstrating that bur roughening is recommended with milled and conventional denture bases bonded to soft liners.

Bur roughening surface treatment resulted in an improvement in SBS for milled and conventional denture base resins relined with a soft liner. This enhancement in bond performance was further validated by SEM analysis of the failure modes, which predominantly exhibited cohesive and mixed failures in Bur roughening specimens.

Based on the findings, bur roughening is recommended as an effective surface treatment for both CAD-CAM milled and conventional denture base resins when used with a soft liner. In contrast, neither sandblasting nor bur roughening is recommended for 3D-printed denture base resins in combination with a soft liner.

For clinical implications, it is recommended that sandblasting be an effective surface treatment for enhancing the bond strength of hard liners to milled, 3D-printed, and conventional denture base resins. In contrast, bur roughening is recommended for improving the bond strength of soft liners with milled and conventional resins. Although ISO 10139-2:2016 (49) specifies that soft long-term denture liners must demonstrate a minimum bond strength of 1.0 MPa in at least 8 out of 10 tested specimens, all groups in this study, whether control, sandblasted, or bur-roughened, exhibited higher bond strength values. However, neither sandblasting nor bur roughening is advisable for 3D-printed denture base resins when used with soft liners.

Limitations of this *in vitro* study include the absence of a complete intraoral environment, such as oral microbiota, salivary pH changes, and occlusal forces. Furthermore, only a 0-degree printing orientation was tested, despite known variations in material properties associated with different printing orientations, and the specimens did not replicate actual denture configurations. Further research is needed, as this study utilized only one type of each liner and examined only two surface treatment methods. Additional investigations employing various types of carbide burs and different sizes of aluminium oxide particles for sandblasting are recommended. In addition to the recommendation of further investigations in condition-simulating oral conditions and *in vivo* studies.

## Conclusion

Based on the findings of this study, sandblasting showed higher SBS values for hard liners bonded to milled, 3D-printed, and conventional denture base resins. Bur roughening demonstrated higher SBS values for soft liners bonded to milled and conventional denture bases. The findings indicate that surface treatment methods can influence the SBS of different denture base materials. Further studies are required to evaluate these effects under clinical conditions and with additional surface treatments and liner materials.

## Data Availability

The original contributions presented in the study are included in the article/Supplementary Material, further inquiries can be directed to the corresponding author.
