# Supplementary figures and images for: The effect of different surface treatment on shear bond strength of soft and hard liners to CAD-CAM and conventional denture base resins: in vitro comparative study
Source: Front Dent Med. 2026 Feb 16;7:1736153. doi: 10.3389/fdmed.2026.1736153 (PMC12950676; doi:10.3389/fdmed.2026.1736153)

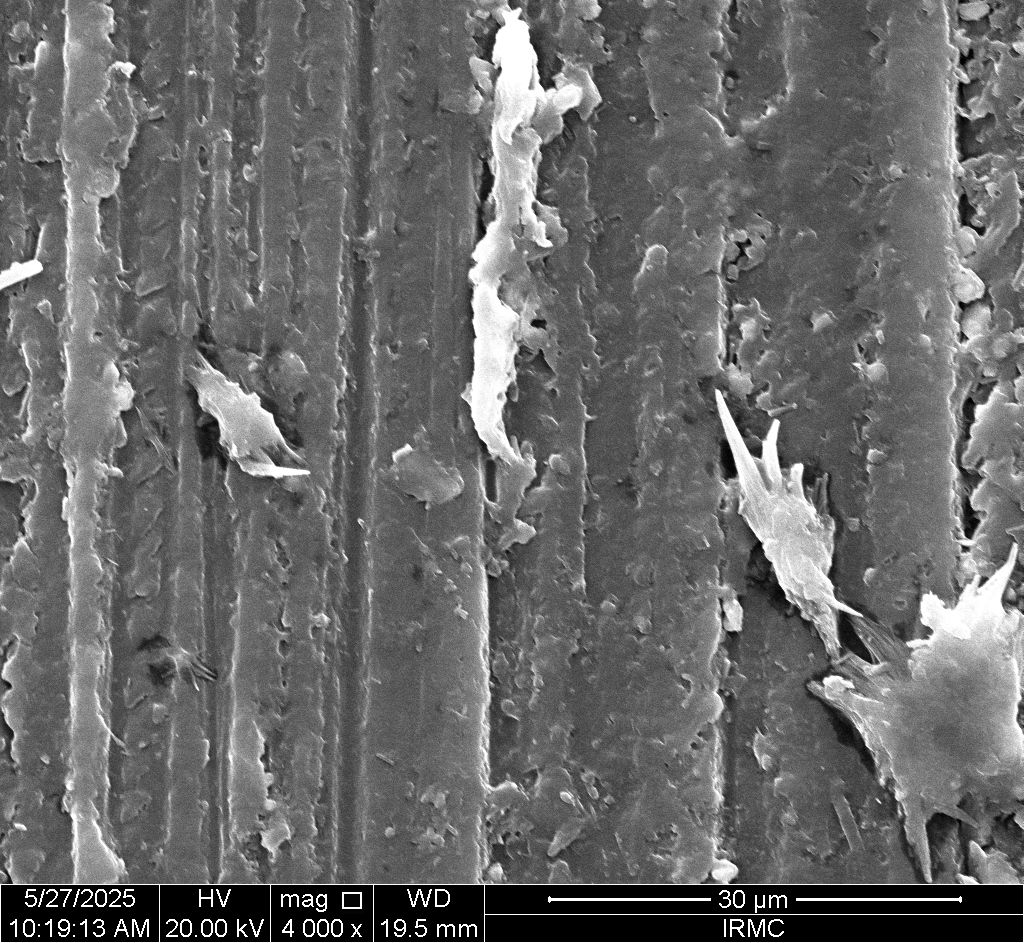

Supplement: Supplementary file 1 [file Datasheet1.zip › Representatives SEM images Show the nature of failure with SEM under x1 - x30/AVADENT ADHESIVE.jpg]

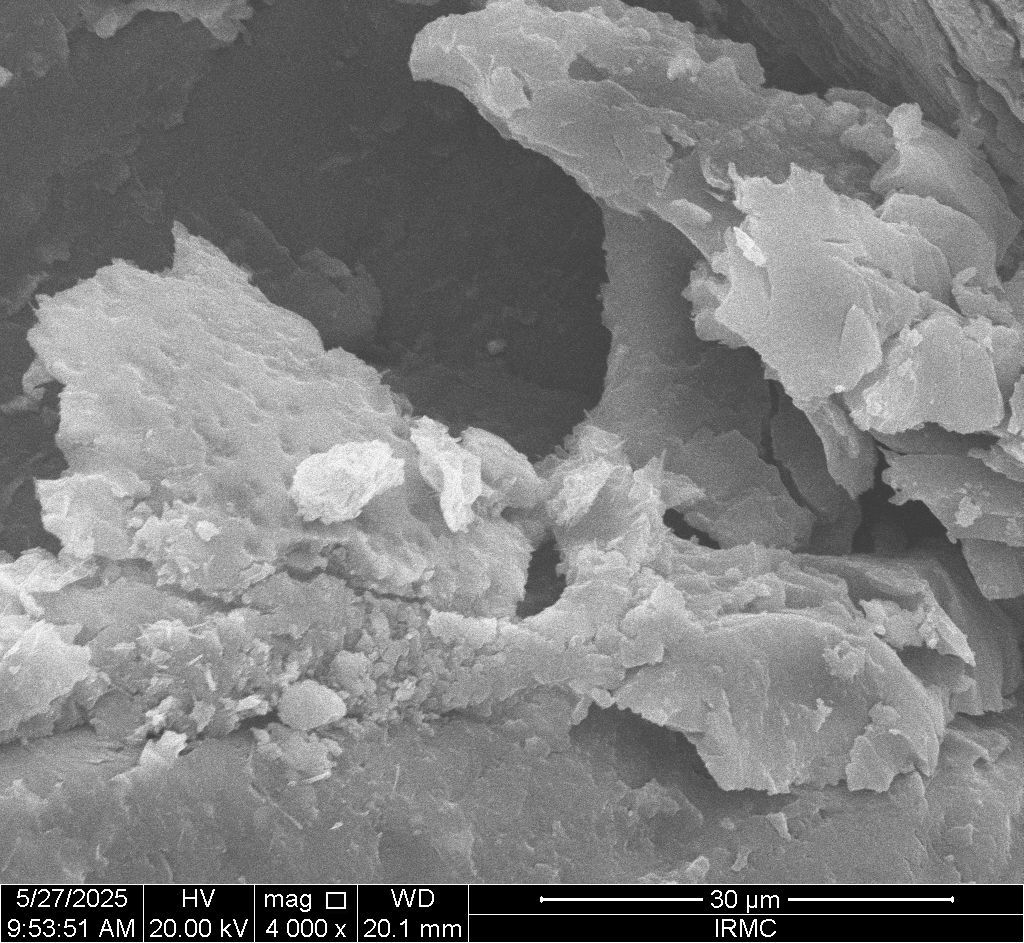

Supplement: Supplementary file 1 [file Datasheet1.zip › Representatives SEM images Show the nature of failure with SEM under x1 - x30/AVADENT COHESIVE.jpg]

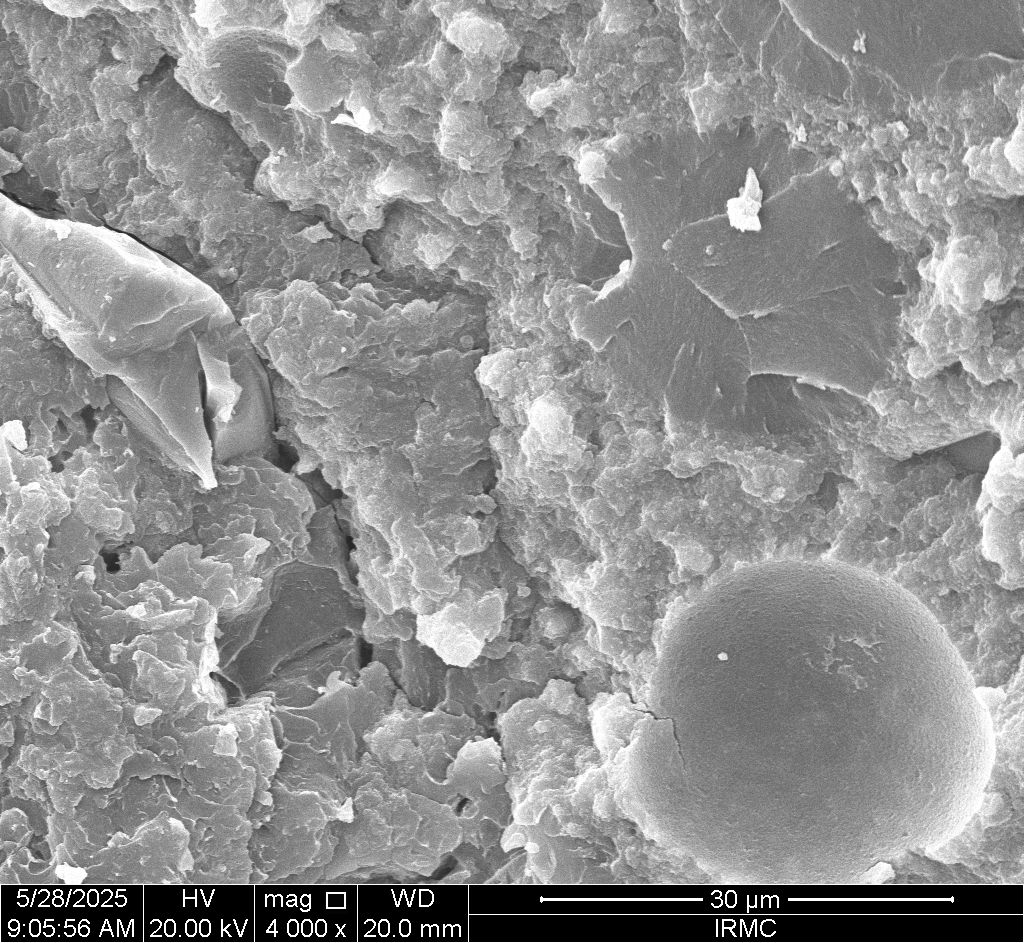

Supplement: Supplementary file 1 [file Datasheet1.zip › Representatives SEM images Show the nature of failure with SEM under x1 - x30/AVCDENT MIXED.jpg]

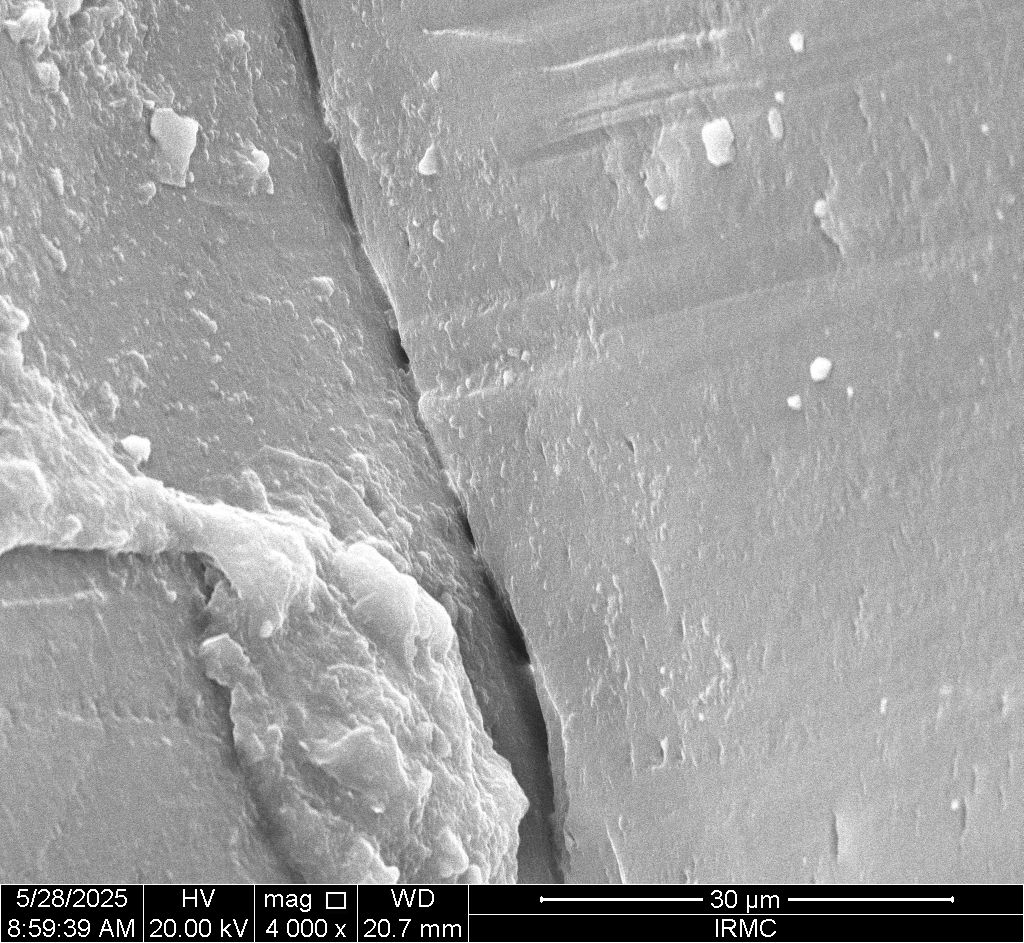

Supplement: Supplementary file 1 [file Datasheet1.zip › Representatives SEM images Show the nature of failure with SEM under x1 - x30/CON COHESIVE.jpg]

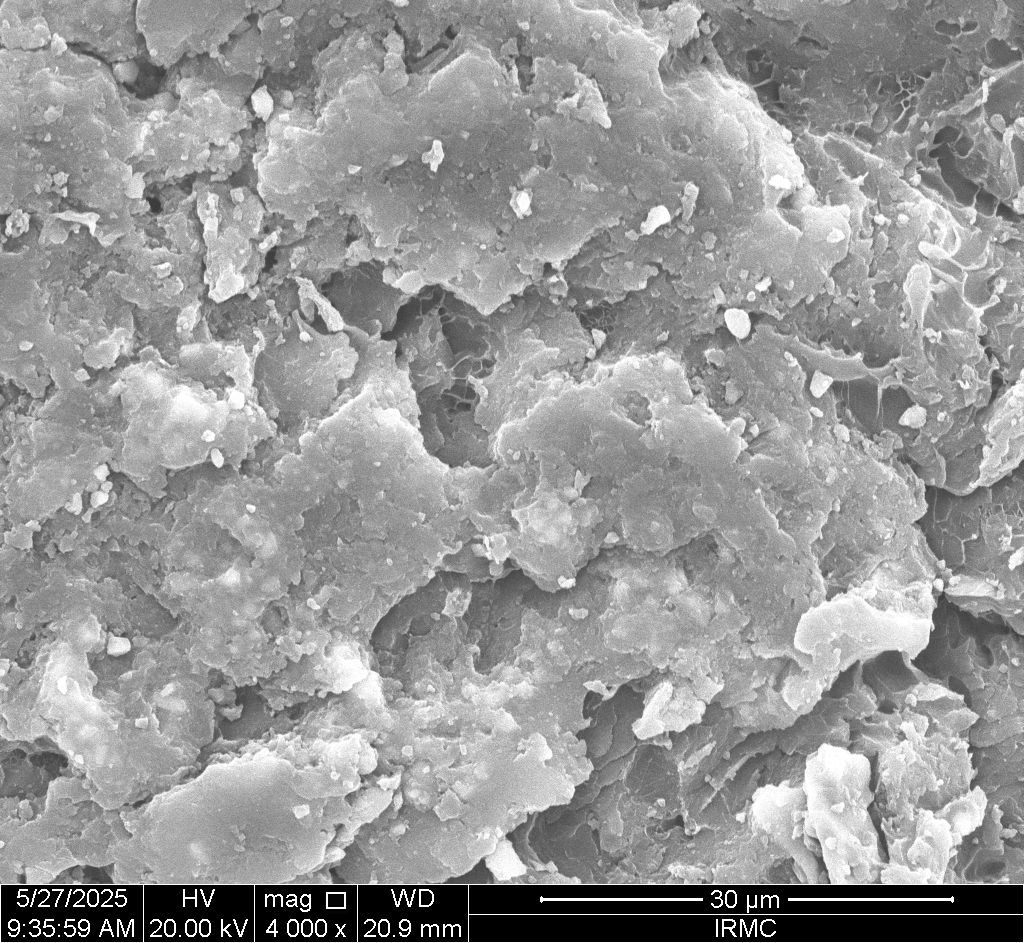

Supplement: Supplementary file 1 [file Datasheet1.zip › Representatives SEM images Show the nature of failure with SEM under x1 - x30/CONVENTIONAL ADHESIVE.jpg]

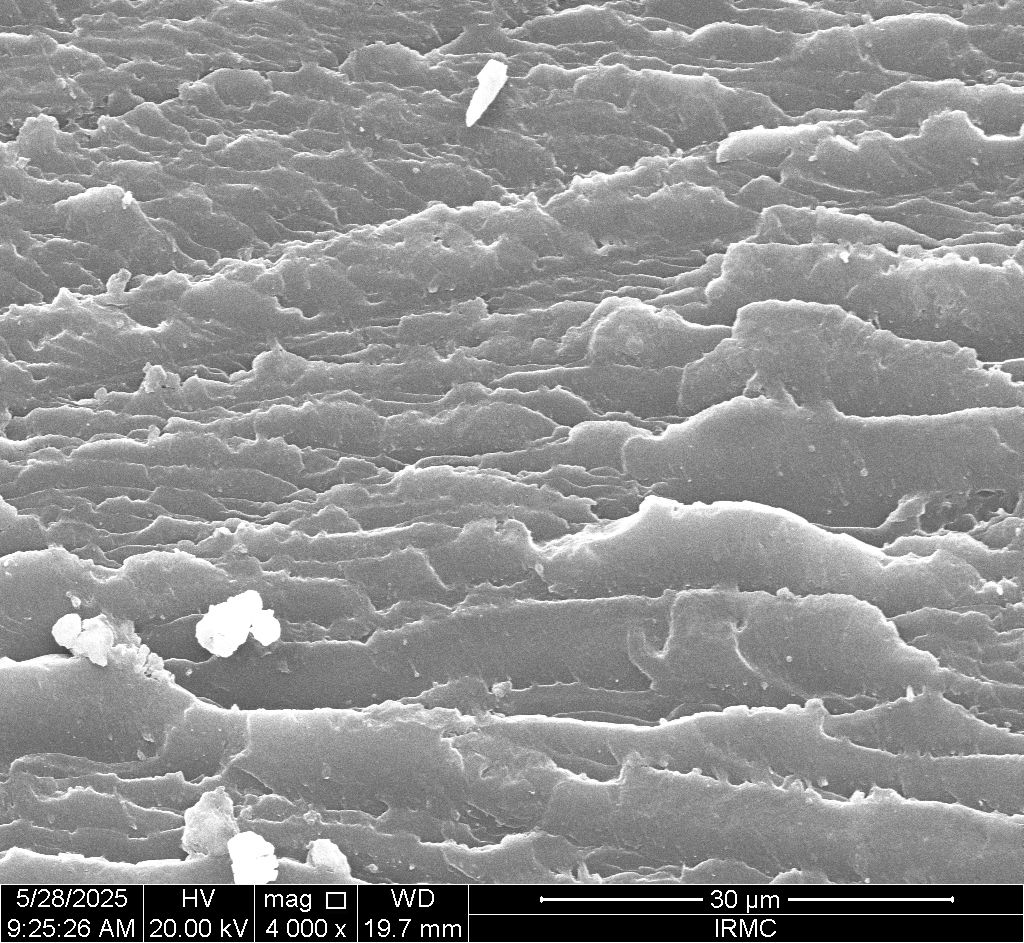

Supplement: Supplementary file 1 [file Datasheet1.zip › Representatives SEM images Show the nature of failure with SEM under x1 - x30/CONVENTIONAL MIXED.jpg]

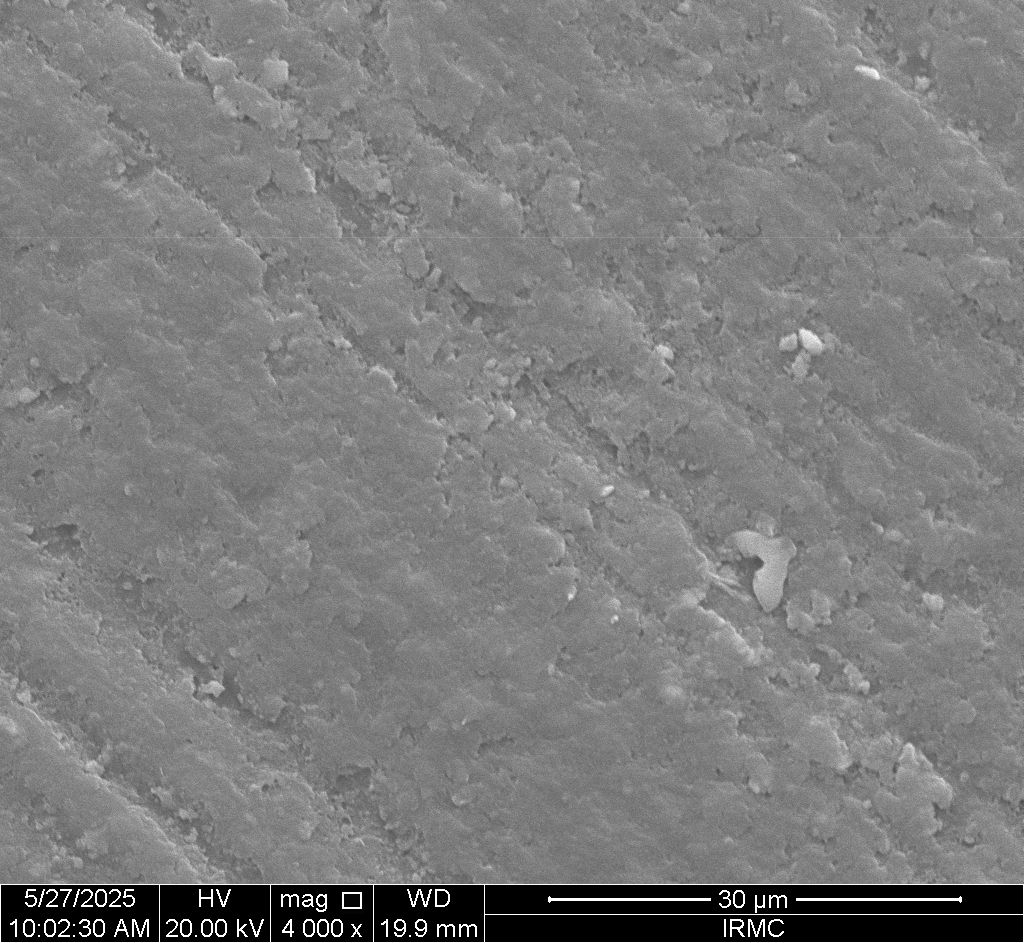

Supplement: Supplementary file 1 [file Datasheet1.zip › Representatives SEM images Show the nature of failure with SEM under x1 - x30/FL ADHESIVE.jpg]

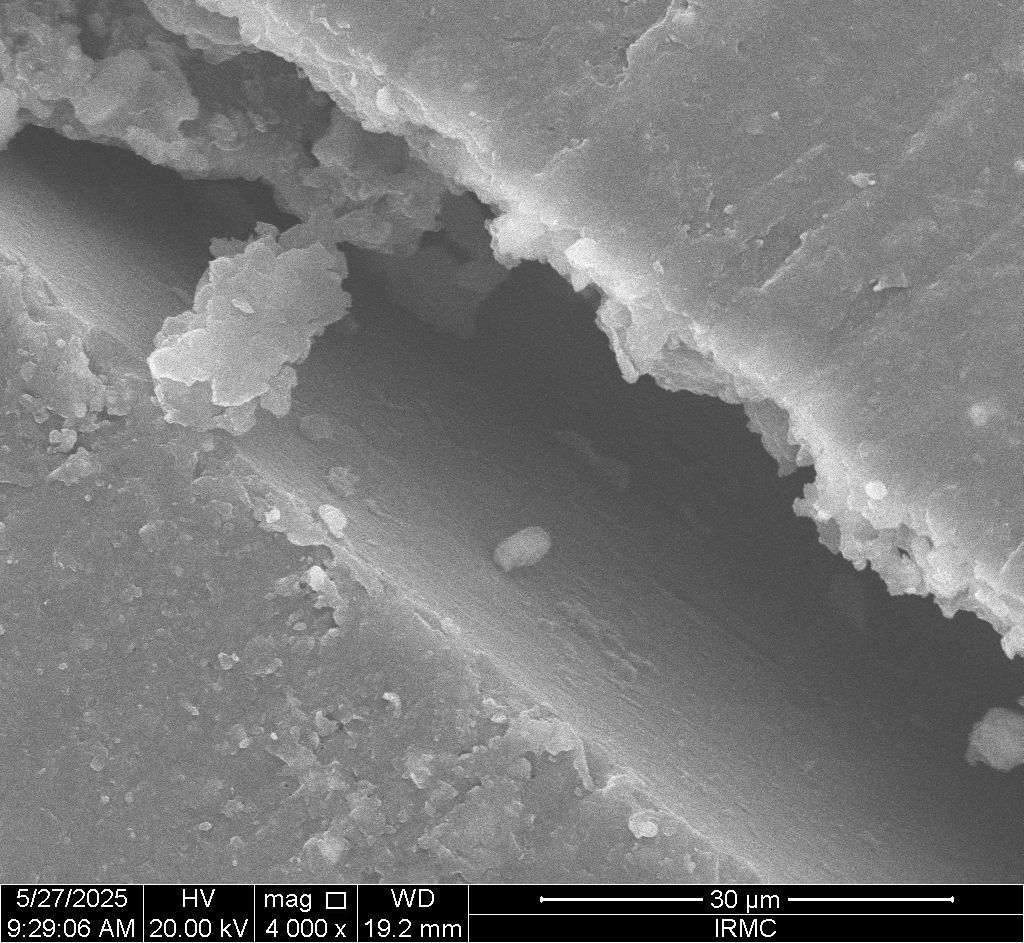

Supplement: Supplementary file 1 [file Datasheet1.zip › Representatives SEM images Show the nature of failure with SEM under x1 - x30/FL- COHESIVE.jpg]

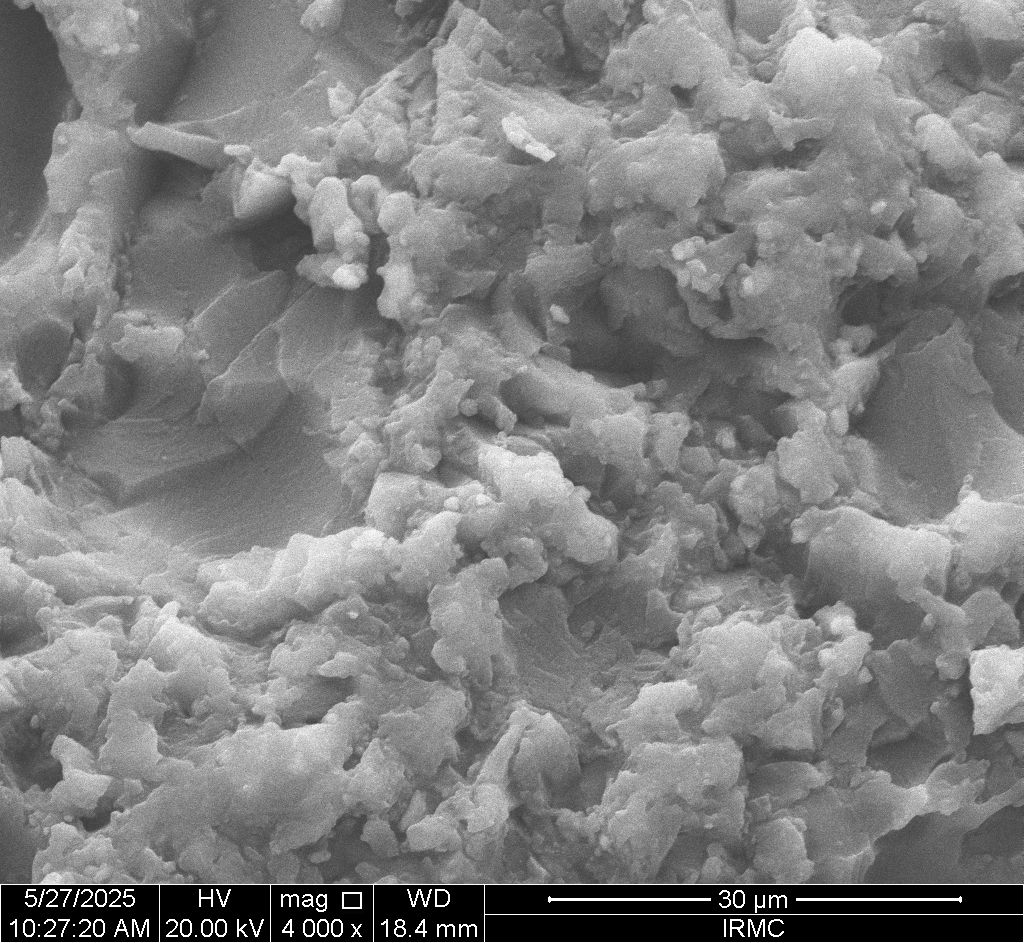

Supplement: Supplementary file 1 [file Datasheet1.zip › Representatives SEM images Show the nature of failure with SEM under x1 - x30/FL MIXED.jpg]

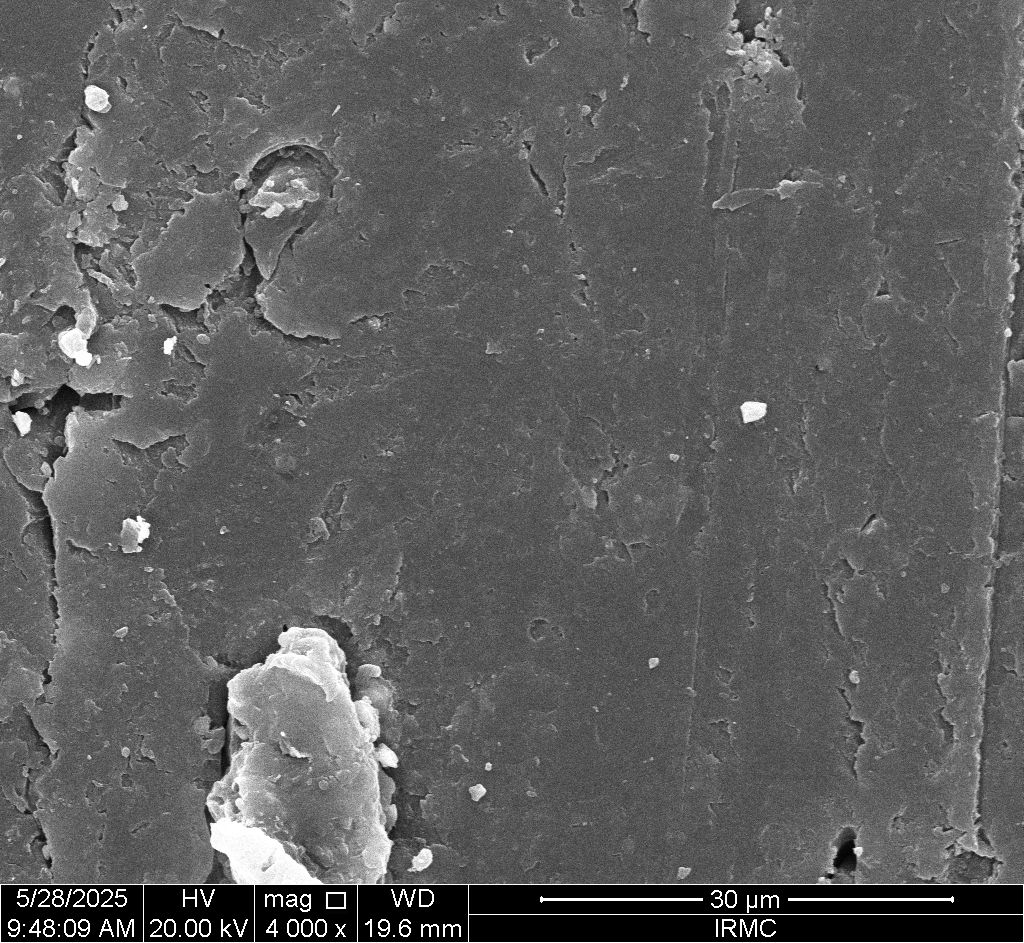

Supplement: Supplementary file 1 [file Datasheet1.zip › Representatives SEM images Show the nature of failure with SEM under x1 - x30/IVOCAD ADHESIVE.jpg]

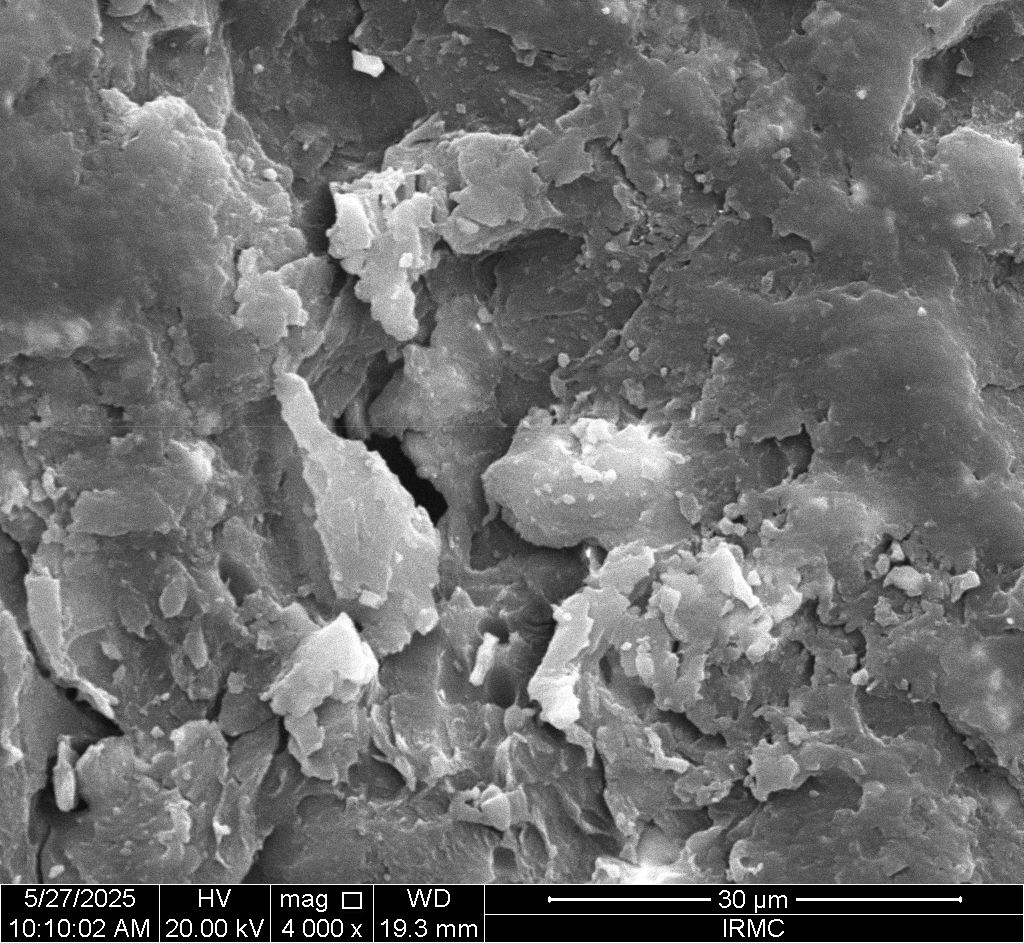

Supplement: Supplementary file 1 [file Datasheet1.zip › Representatives SEM images Show the nature of failure with SEM under x1 - x30/IVOCAD COHESIVE.jpg]

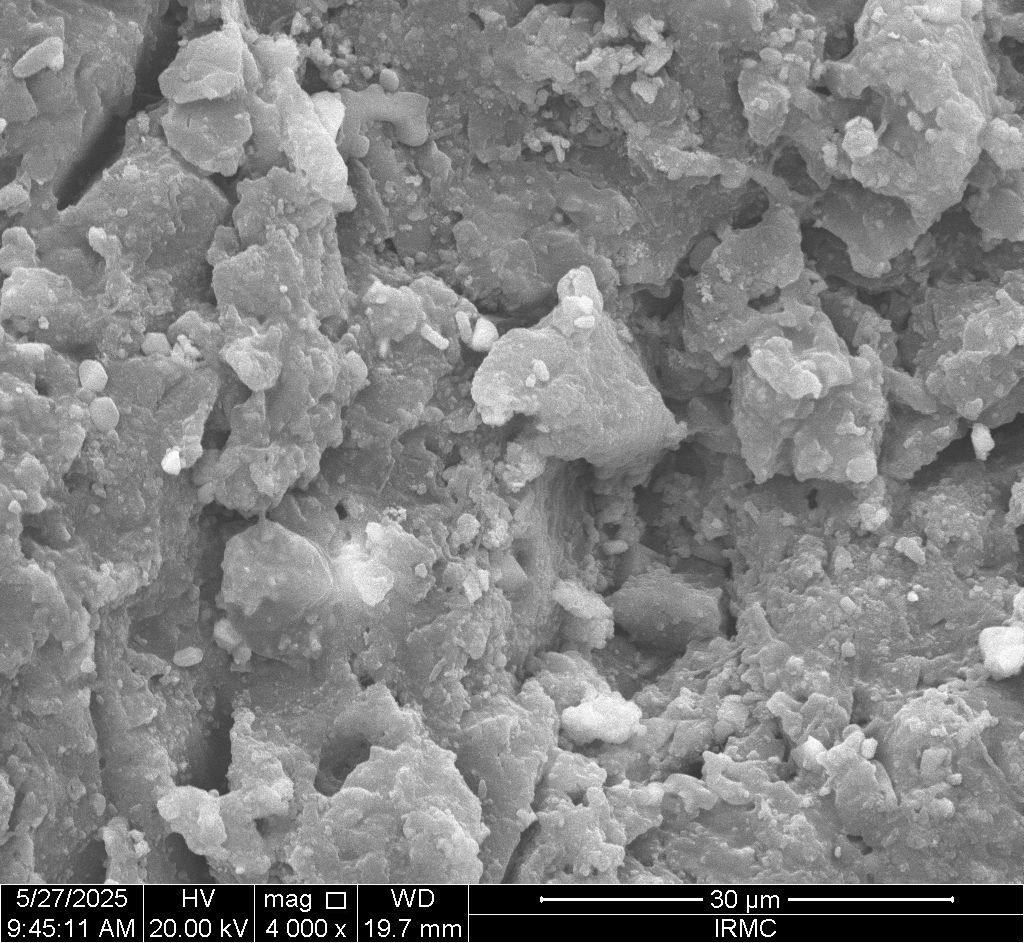

Supplement: Supplementary file 1 [file Datasheet1.zip › Representatives SEM images Show the nature of failure with SEM under x1 - x30/IVOCAD MIXED.jpg]

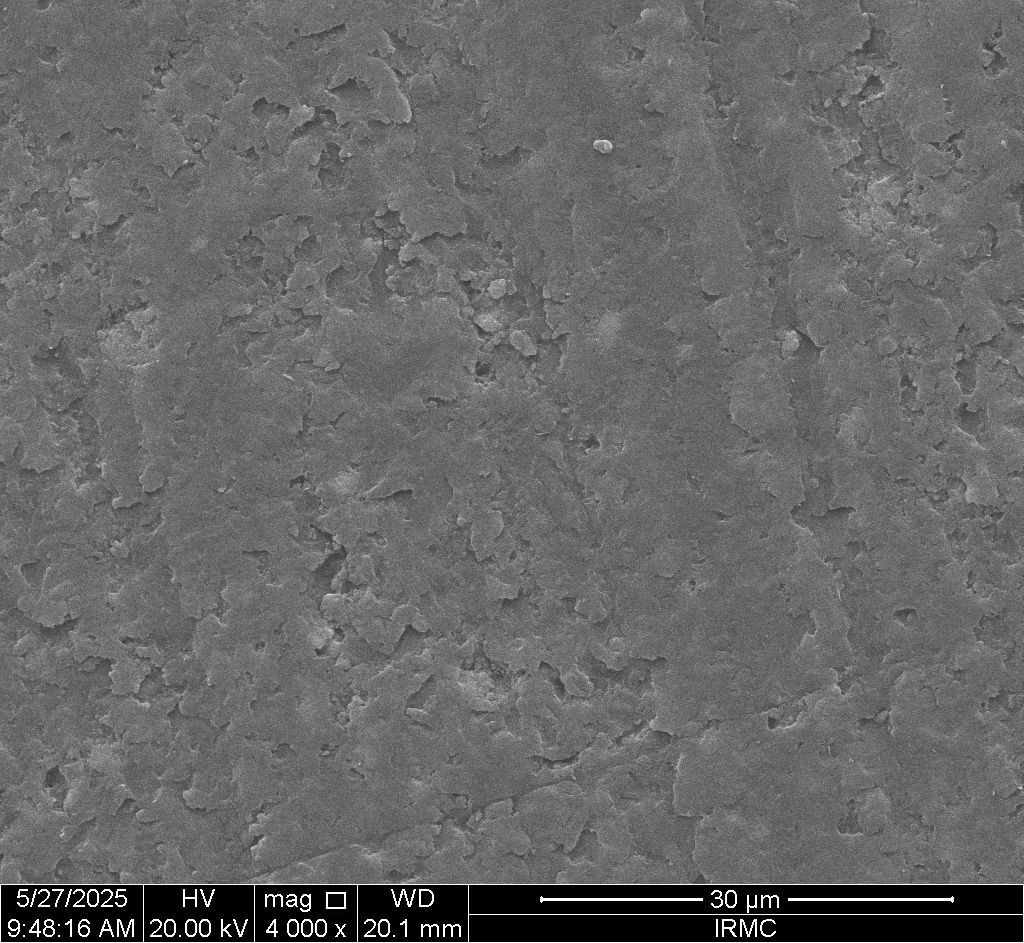

Supplement: Supplementary file 1 [file Datasheet1.zip › Representatives SEM images Show the nature of failure with SEM under x1 - x30/ND ADHESIVE.jpg]

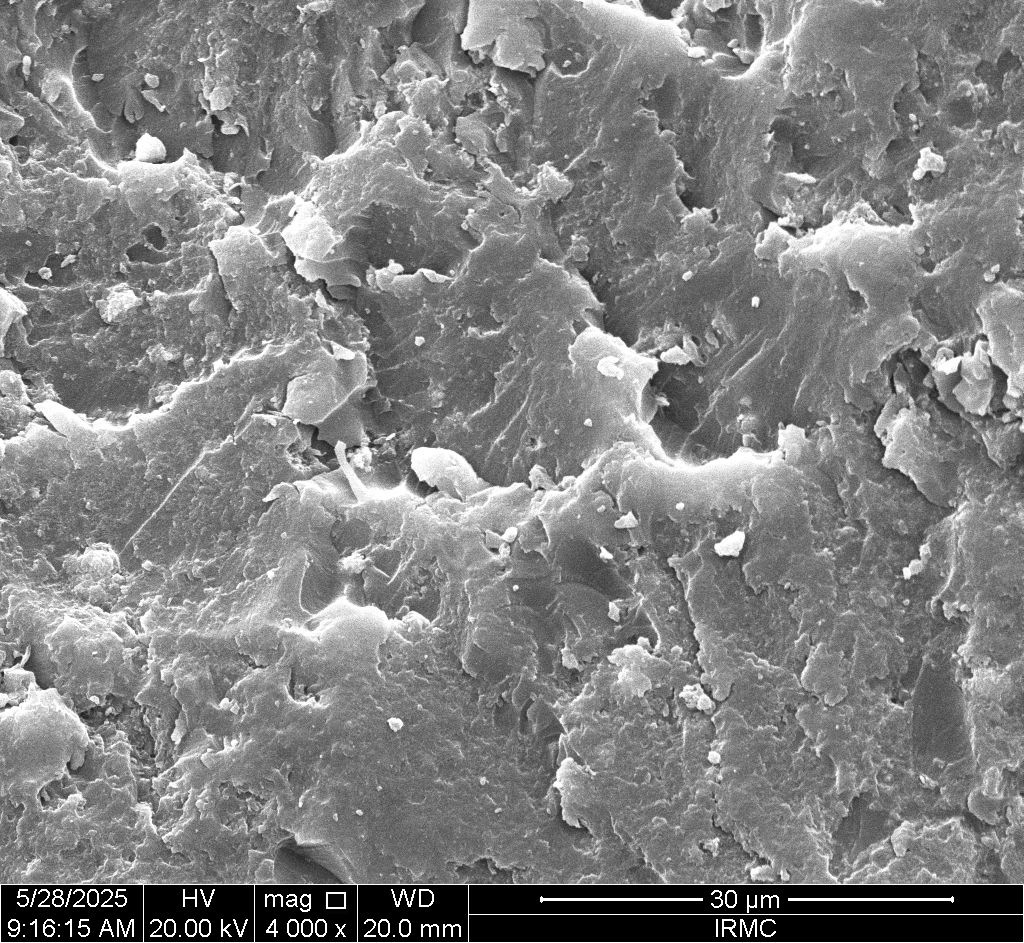

Supplement: Supplementary file 1 [file Datasheet1.zip › Representatives SEM images Show the nature of failure with SEM under x1 - x30/ND COHESIVE.jpg]

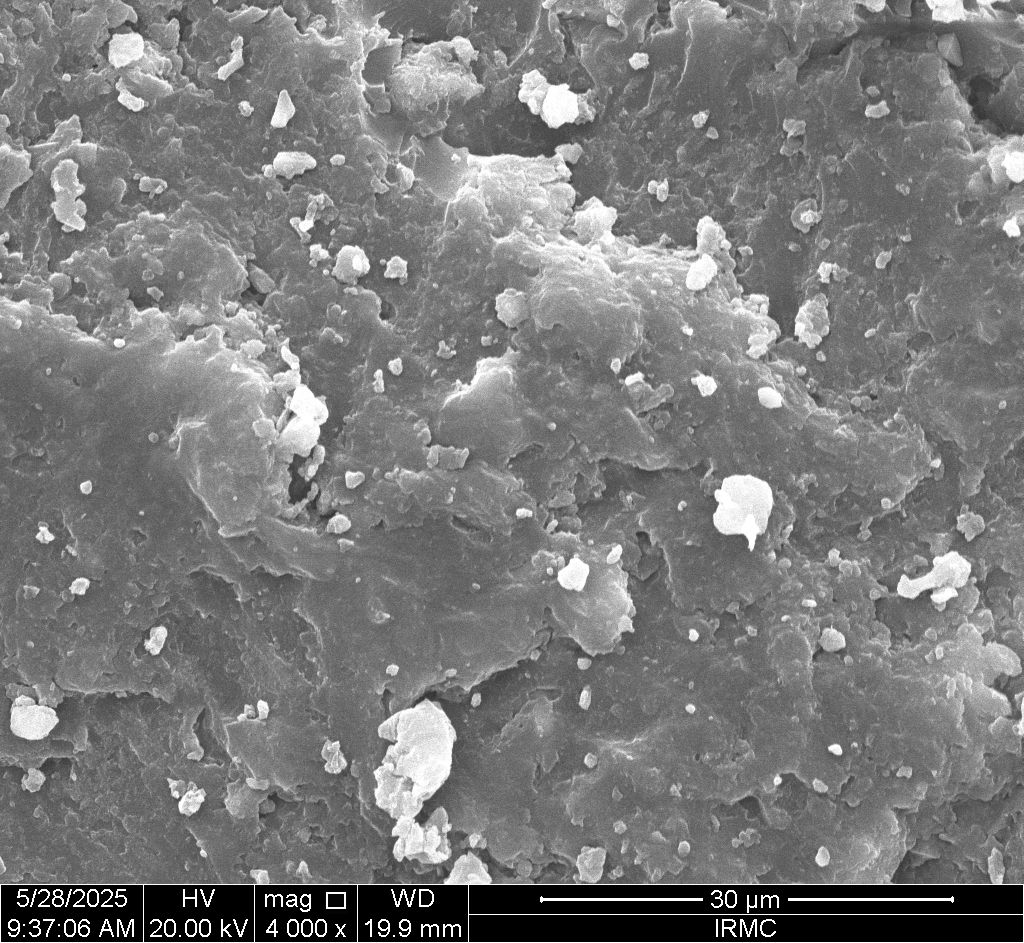

Supplement: Supplementary file 1 [file Datasheet1.zip › Representatives SEM images Show the nature of failure with SEM under x1 - x30/ND MIXED.jpg]

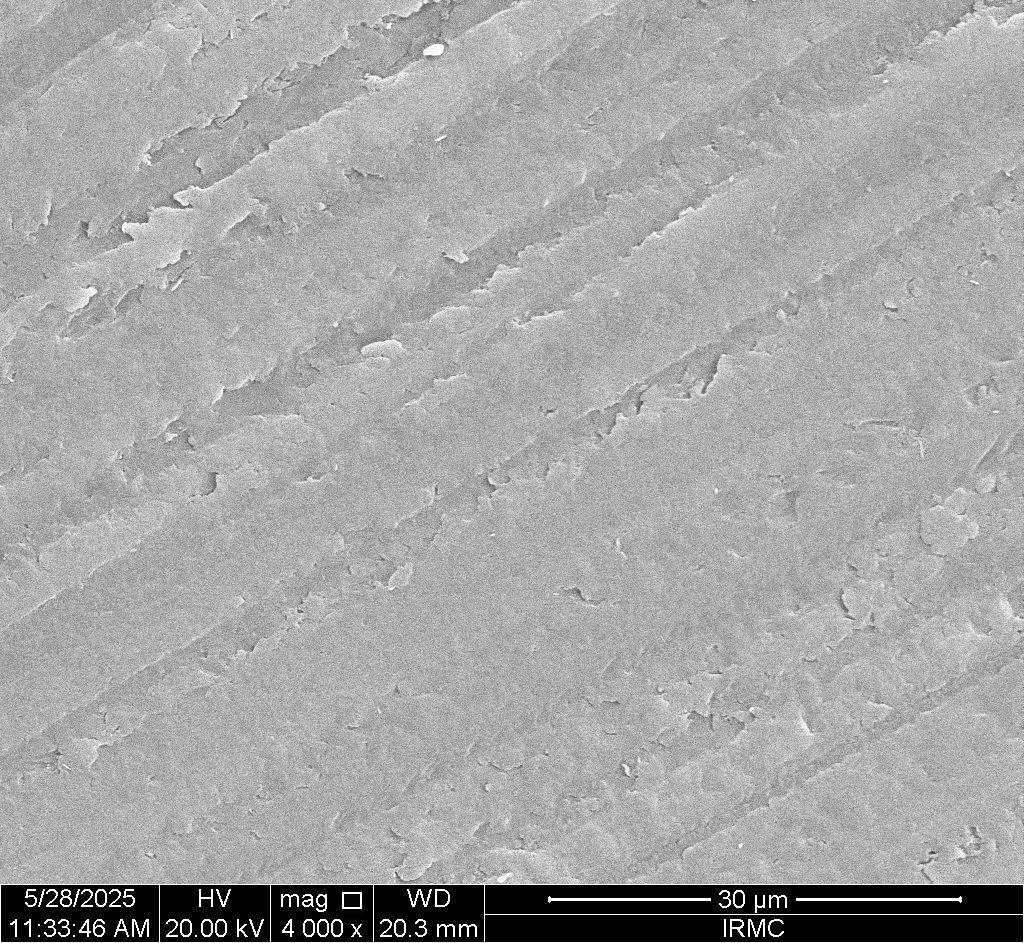

Supplement: Supplementary file 2 [file Datasheet2.zip › Different surface treatment under SEM under x30/AVADENT CONTROL.jpg]

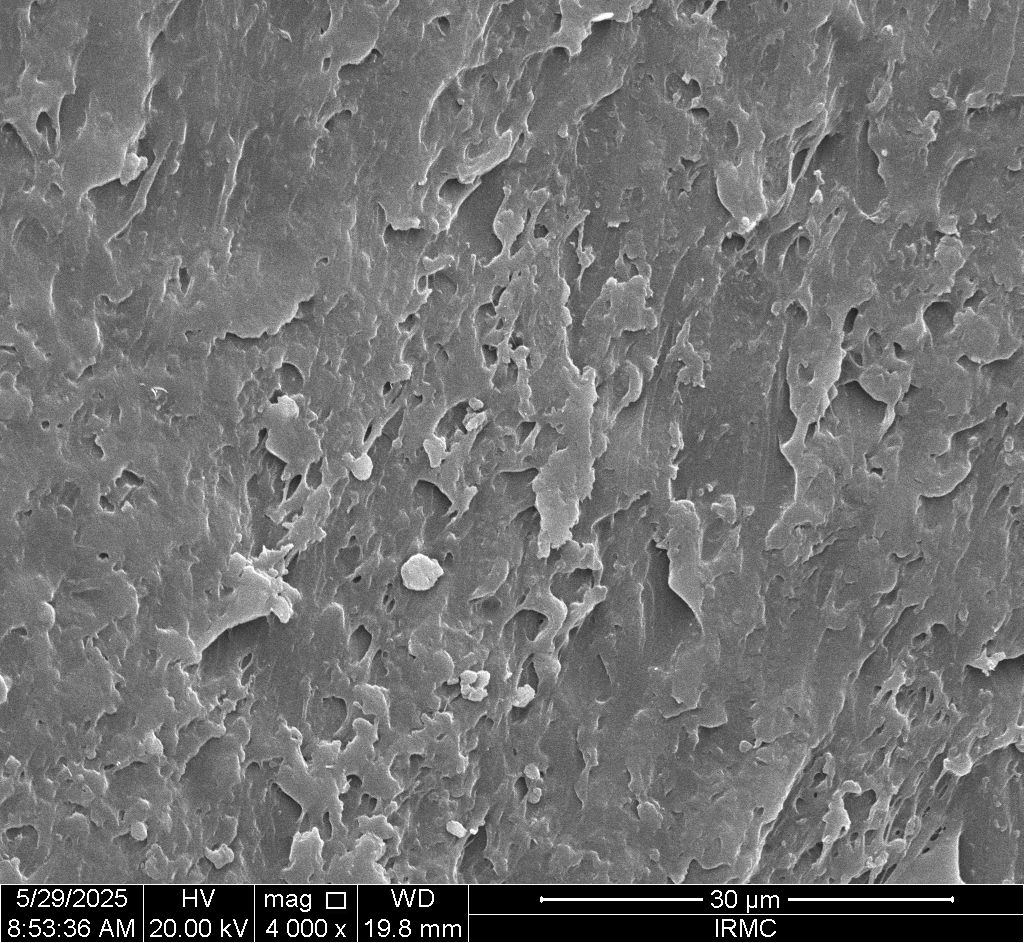

Supplement: Supplementary file 2 [file Datasheet2.zip › Different surface treatment under SEM under x30/AVADENT RB.jpg]

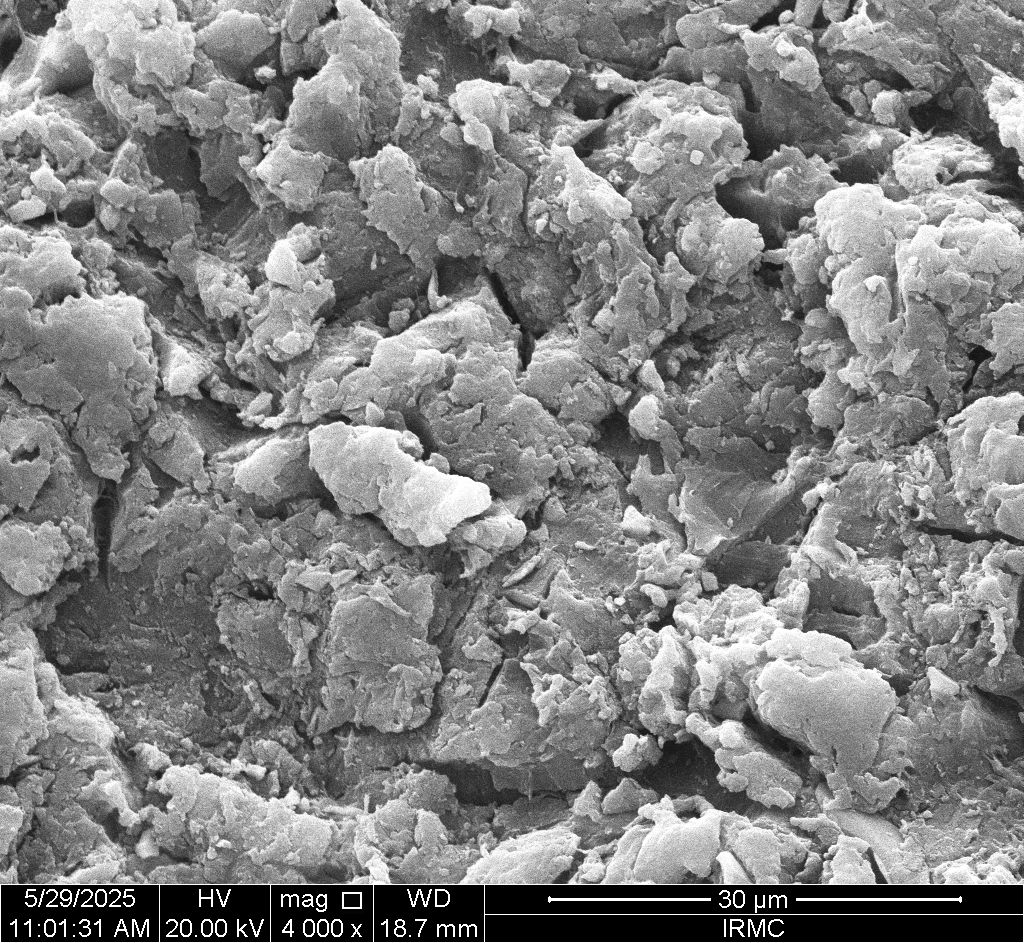

Supplement: Supplementary file 2 [file Datasheet2.zip › Different surface treatment under SEM under x30/AVADENT SB.jpg]

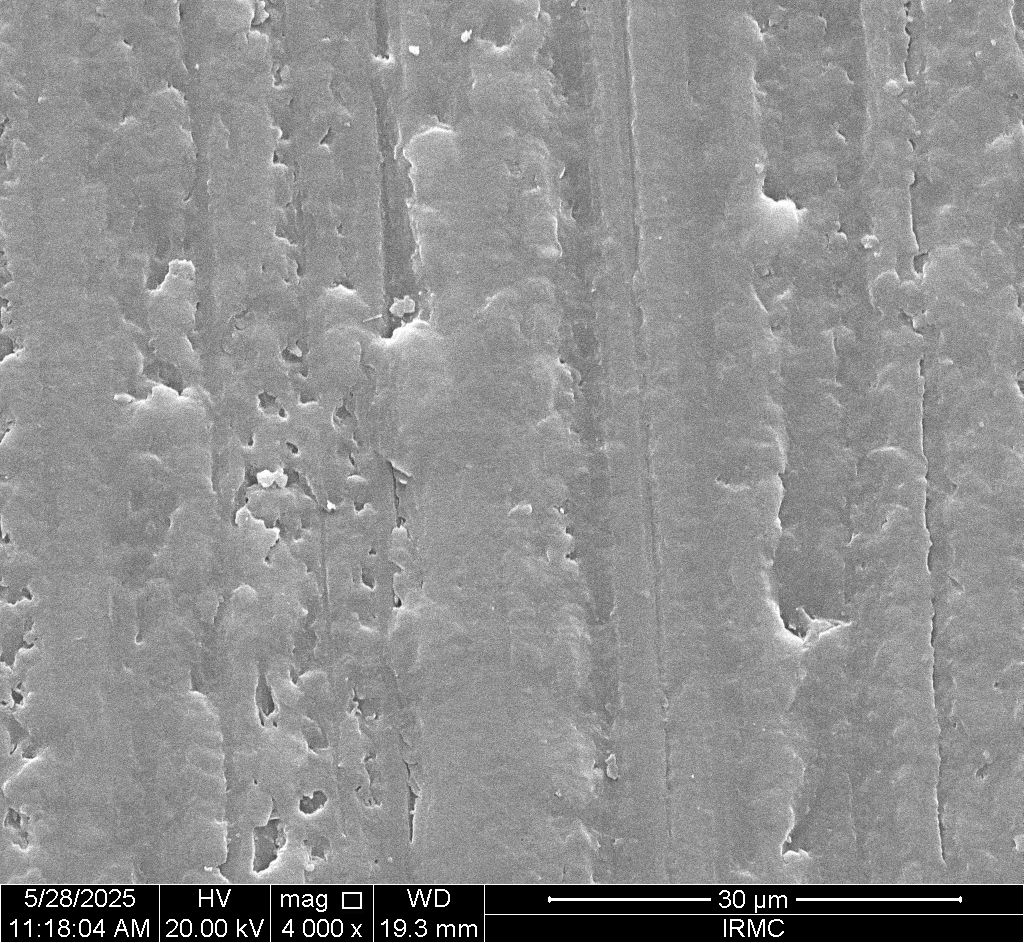

Supplement: Supplementary file 2 [file Datasheet2.zip › Different surface treatment under SEM under x30/CONVENTIONAL CONTROL.jpg]

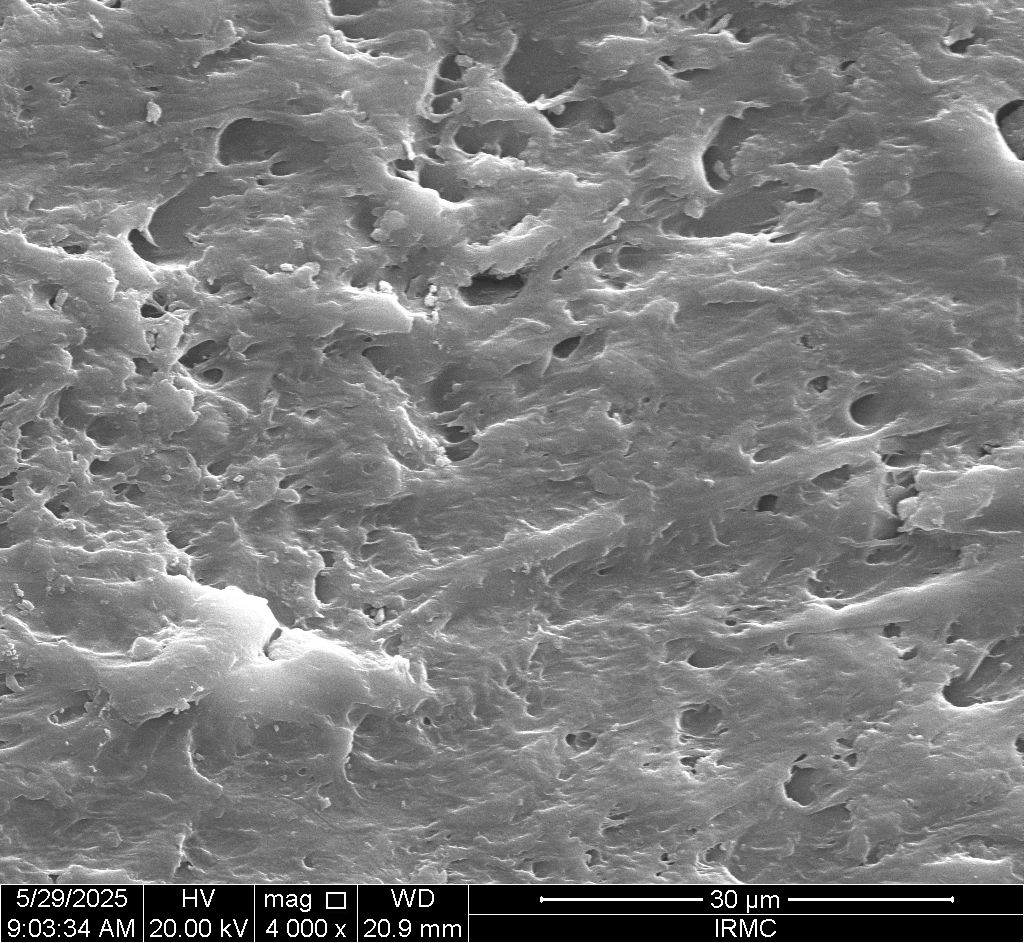

Supplement: Supplementary file 2 [file Datasheet2.zip › Different surface treatment under SEM under x30/CONVENTIONAL RB.jpg]

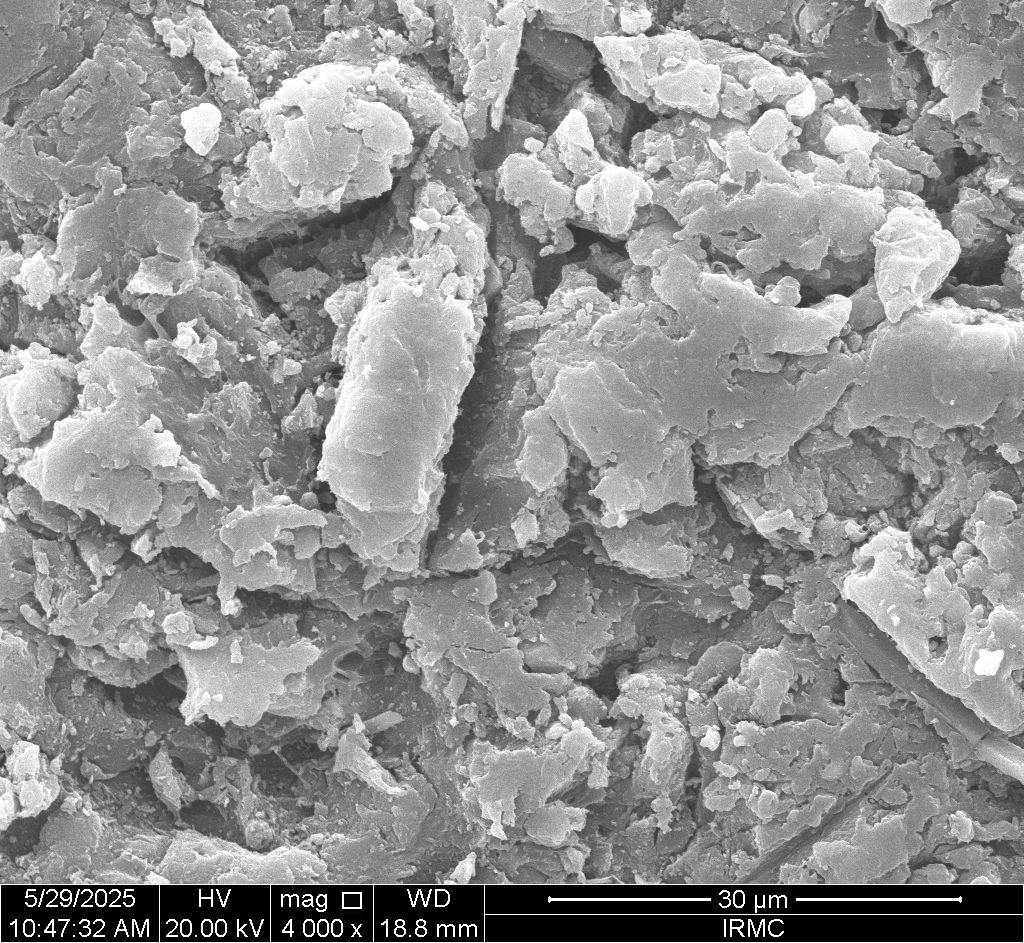

Supplement: Supplementary file 2 [file Datasheet2.zip › Different surface treatment under SEM under x30/CONVENTIONAL SB.jpg]

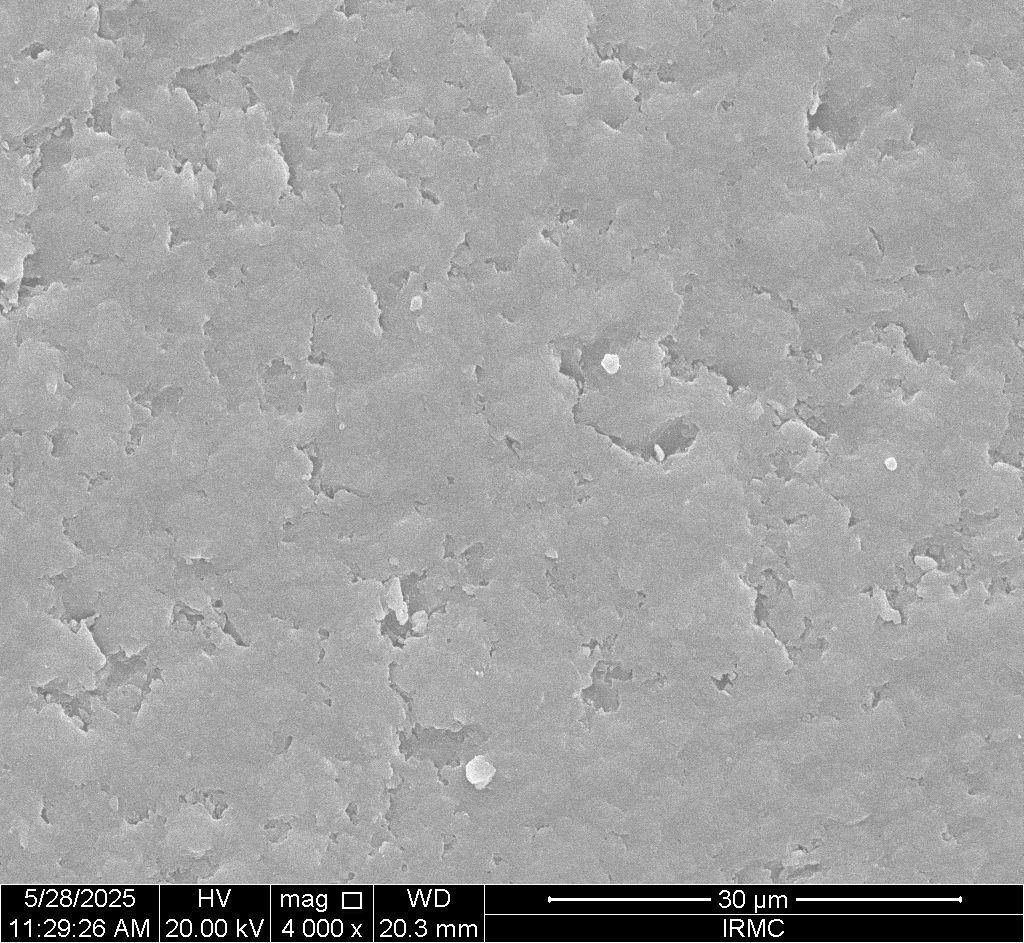

Supplement: Supplementary file 2 [file Datasheet2.zip › Different surface treatment under SEM under x30/FL CONTROL.jpg]

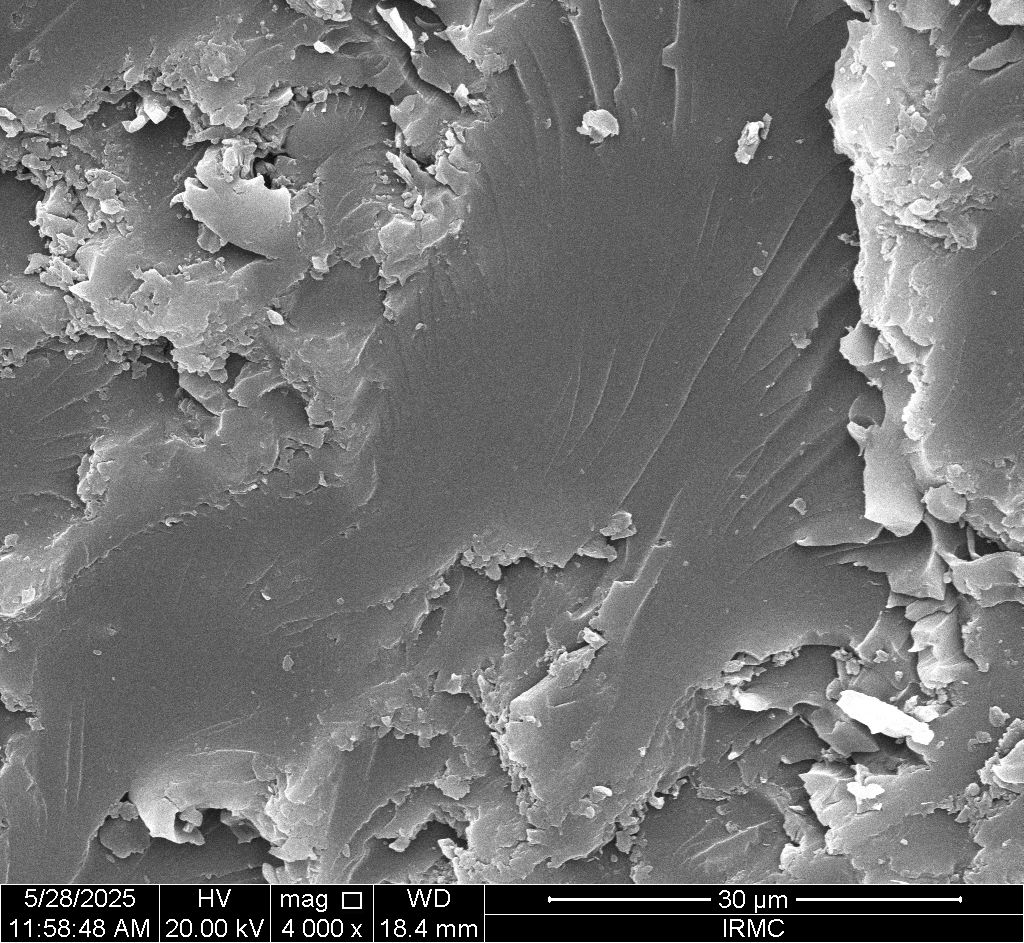

Supplement: Supplementary file 2 [file Datasheet2.zip › Different surface treatment under SEM under x30/FL RB.jpg]

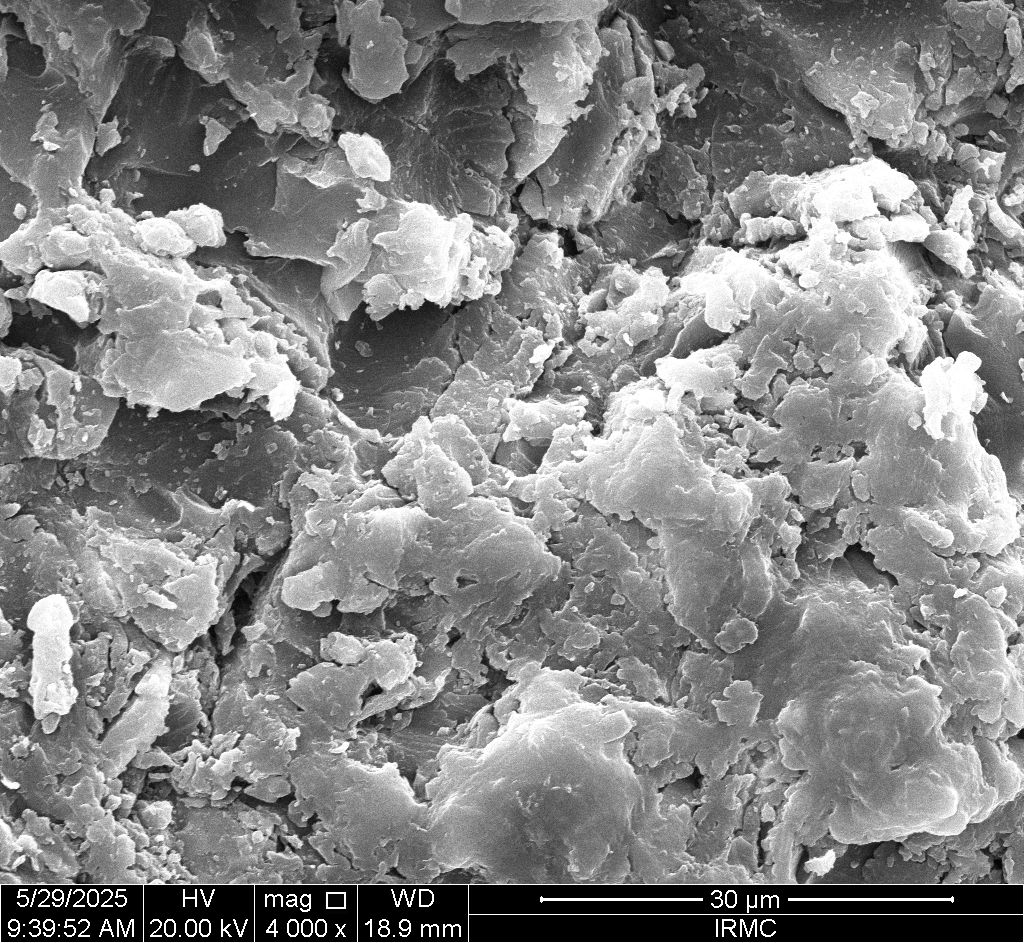

Supplement: Supplementary file 2 [file Datasheet2.zip › Different surface treatment under SEM under x30/FL SB.jpg]

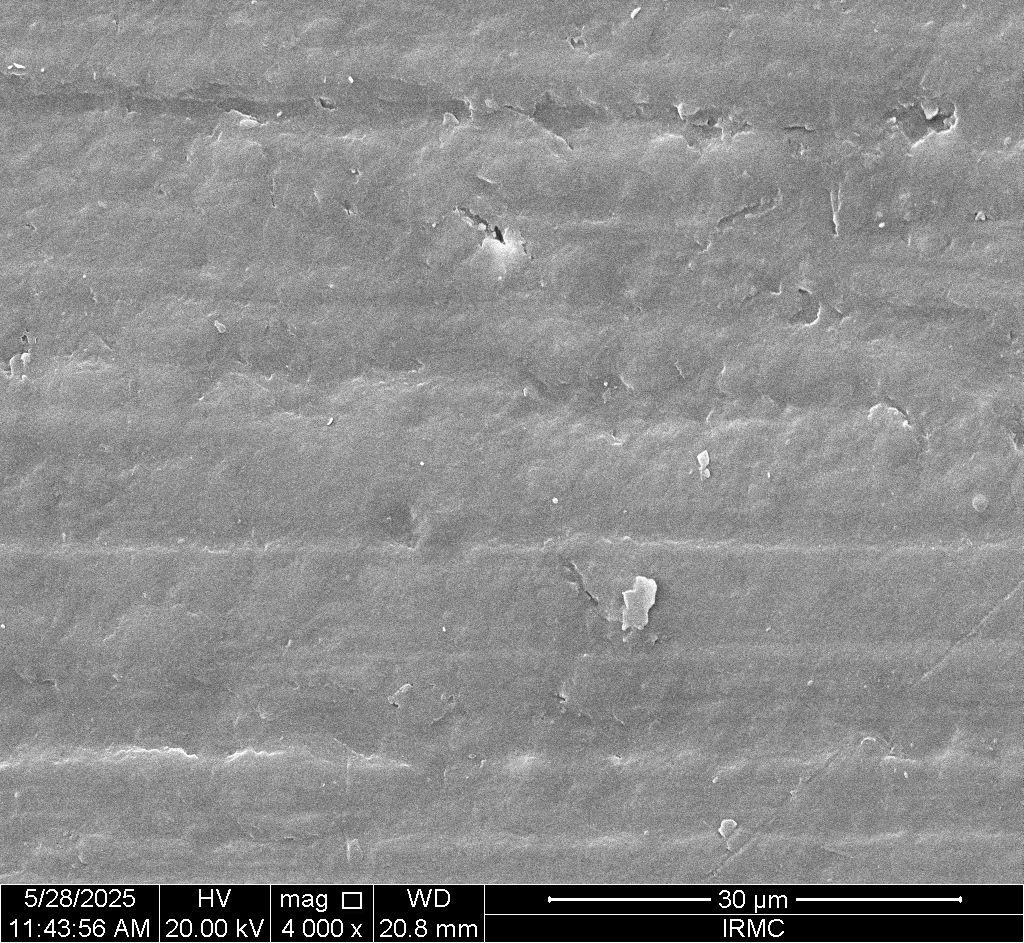

Supplement: Supplementary file 2 [file Datasheet2.zip › Different surface treatment under SEM under x30/IVO CONTROL.jpg]

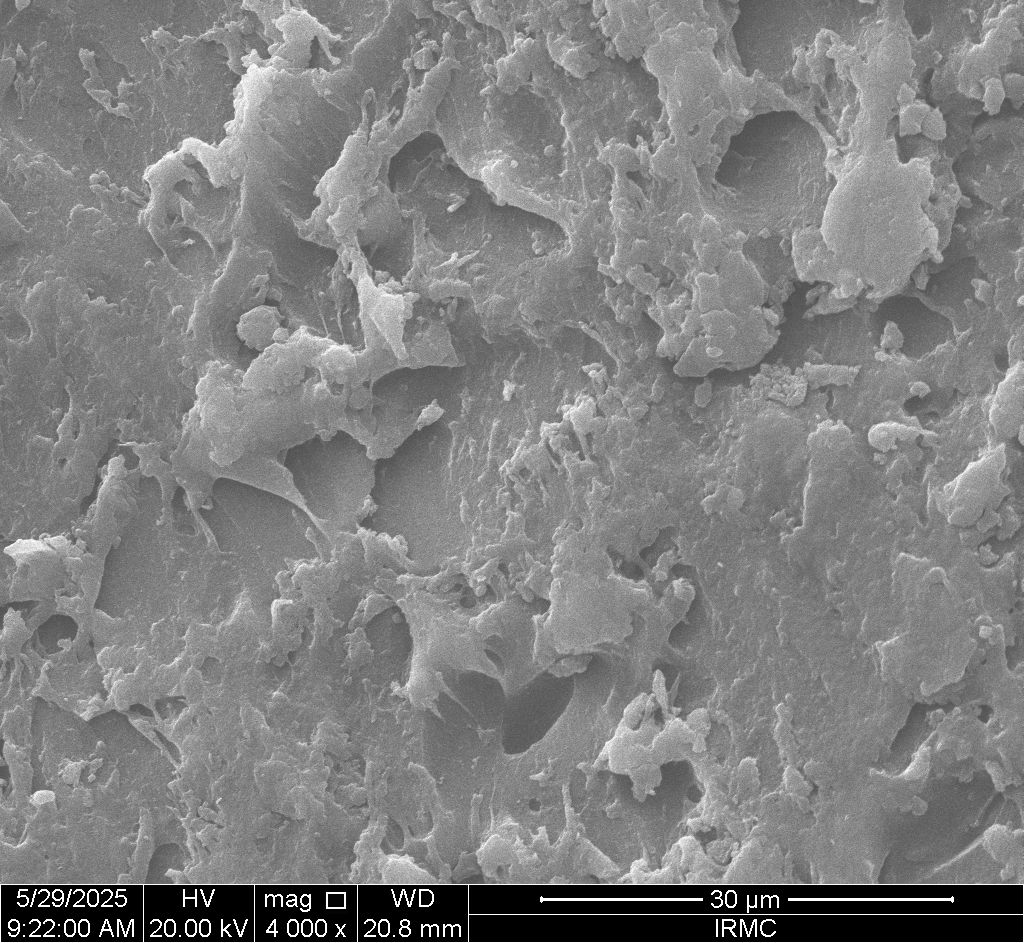

Supplement: Supplementary file 2 [file Datasheet2.zip › Different surface treatment under SEM under x30/IVOCAD RB.jpg]

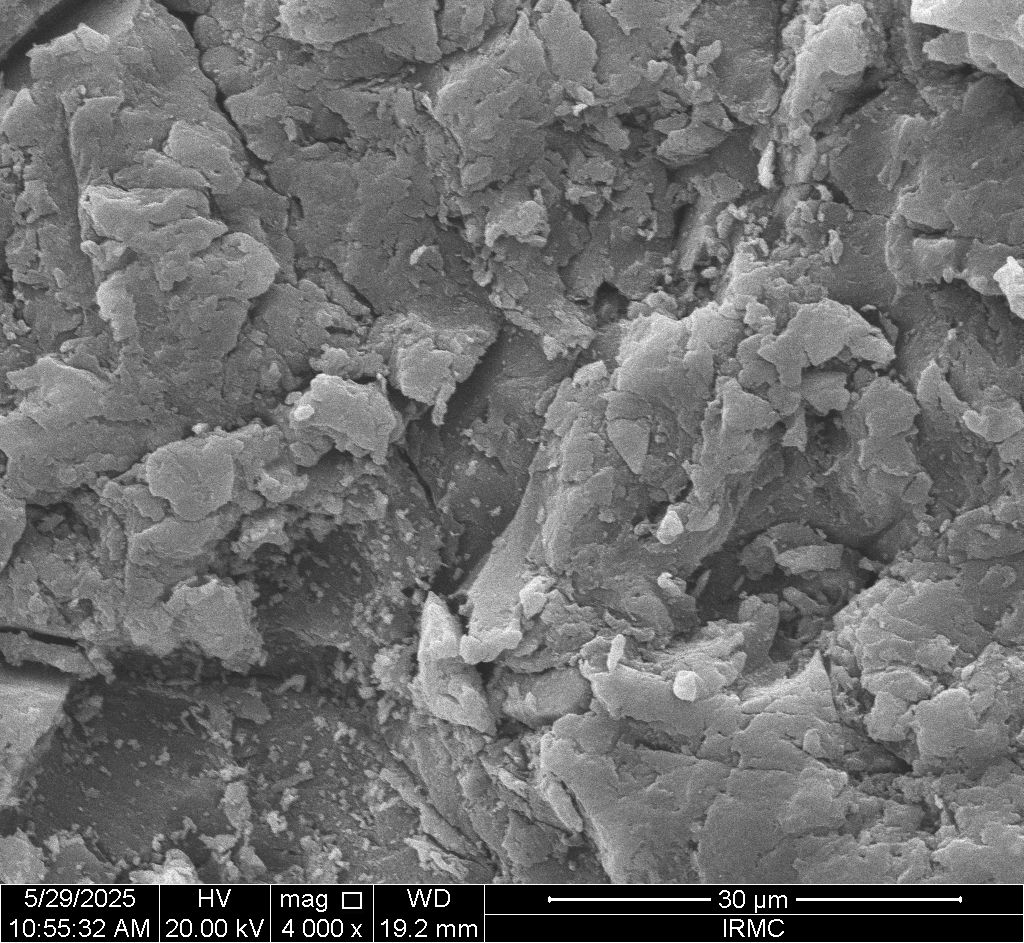

Supplement: Supplementary file 2 [file Datasheet2.zip › Different surface treatment under SEM under x30/IVOCAD SB.jpg]

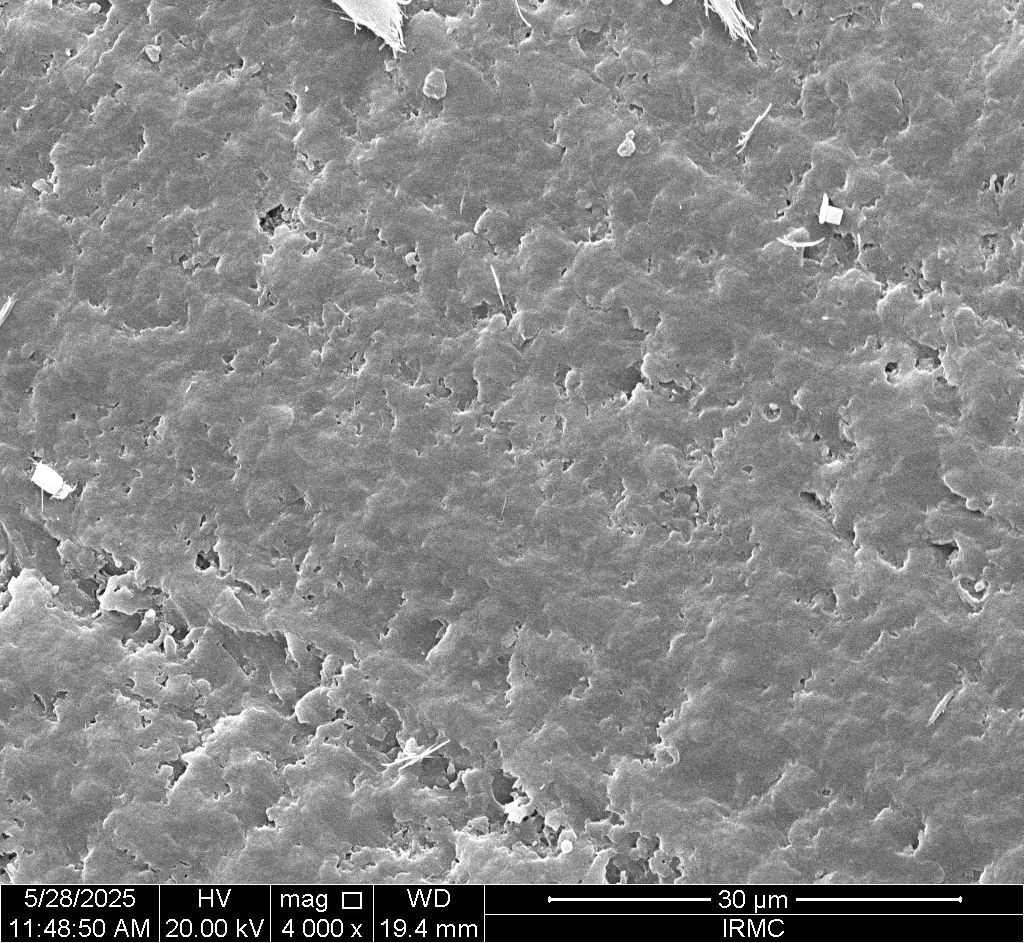

Supplement: Supplementary file 2 [file Datasheet2.zip › Different surface treatment under SEM under x30/ND CONTROL.jpg]

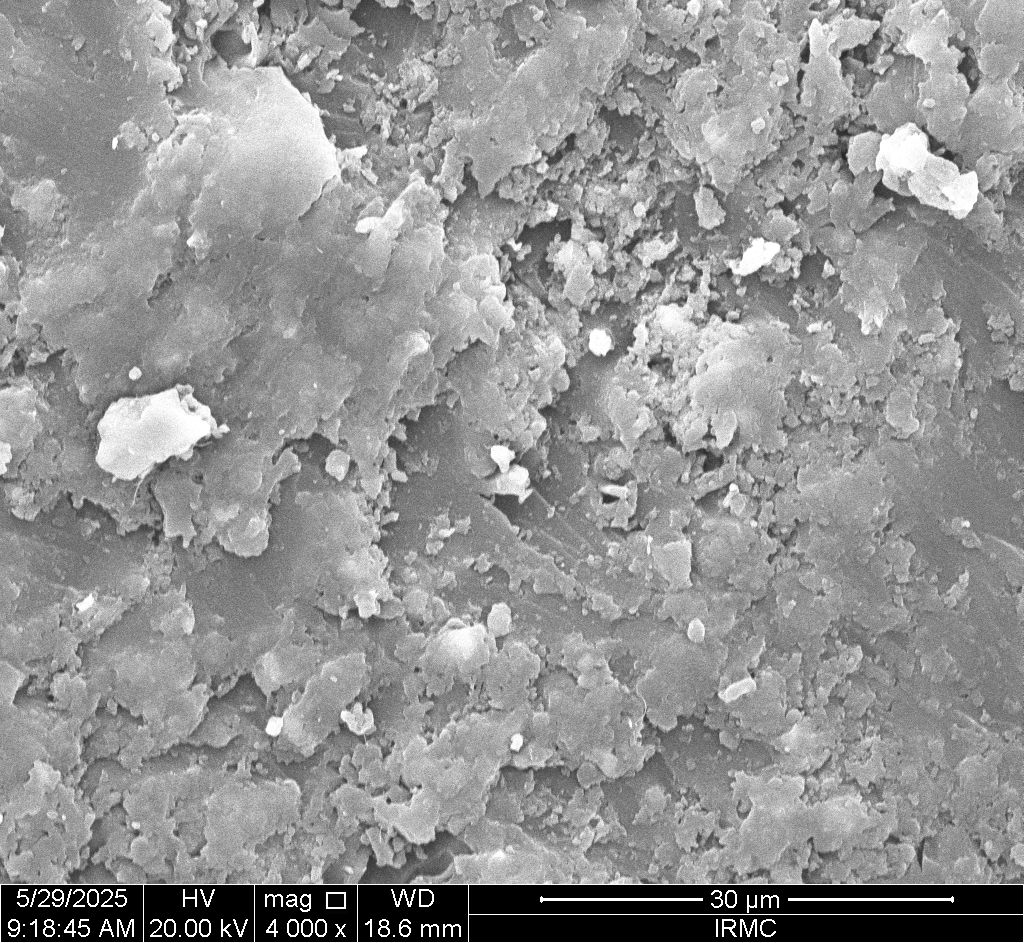

Supplement: Supplementary file 2 [file Datasheet2.zip › Different surface treatment under SEM under x30/ND RB.jpg]

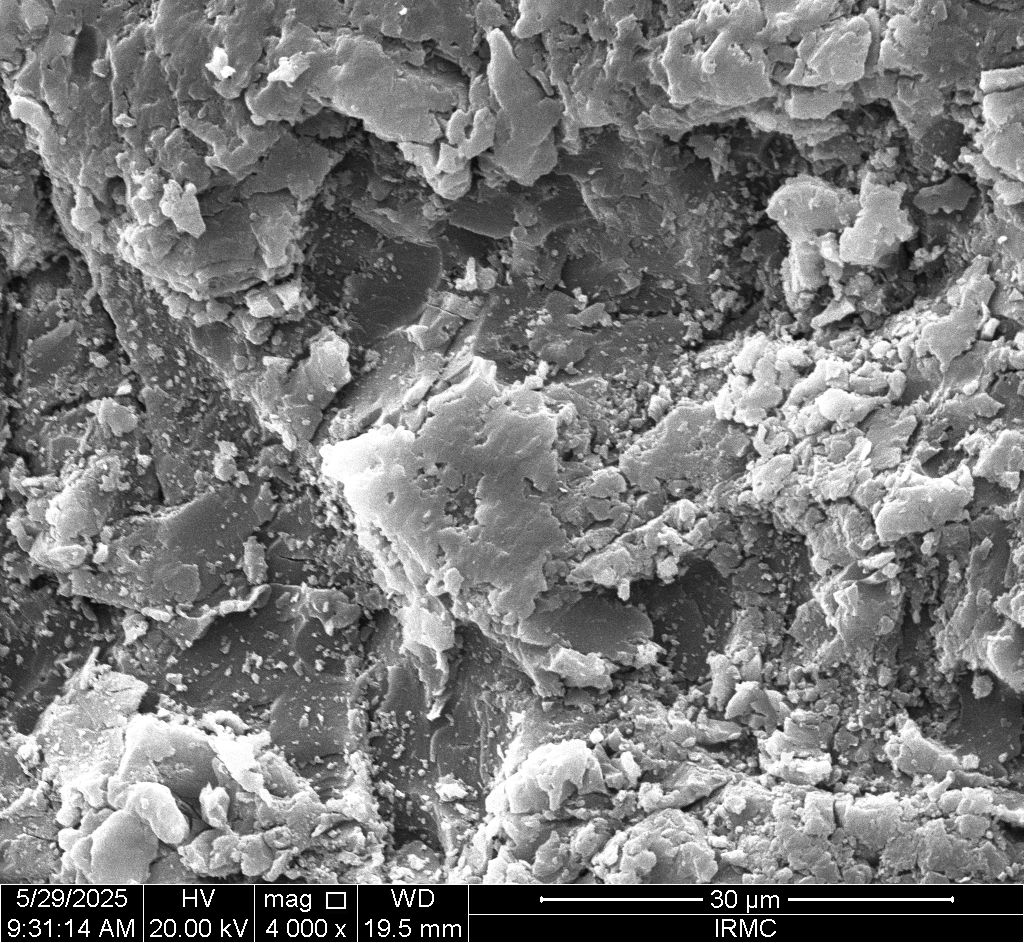

Supplement: Supplementary file 2 [file Datasheet2.zip › Different surface treatment under SEM under x30/ND SB.jpg]
